# Stromal Fibroblasts Counteract the Caveolin-1-Dependent Radiation Response of LNCaP Prostate Carcinoma Cells

**DOI:** 10.3389/fonc.2022.802482

**Published:** 2022-01-26

**Authors:** Alina Wittka, Julia Ketteler, Lars Borgards, Patrick Maier, Carsten Herskind, Verena Jendrossek, Diana Klein

**Affiliations:** ^1^Institute of Cell Biology (Cancer Research), University of Duisburg-Essen, Medical Faculty Essen, Essen, Germany; ^2^Department of Radiation Oncology, University Medical Center Mannheim, Medical Faculty Mannheim, Heidelberg University, Mannheim, Germany

**Keywords:** caveolin-1, tumor microenvironment, fibroblast, dasatinib, radiotherapy, therapy resistance, prostate cancer, SRC

## Abstract

In prostate cancer (PCa), a characteristic stromal–epithelial redistribution of the membrane protein caveolin 1 (CAV1) occurs upon tumor progression, where a gain of CAV1 in the malignant epithelial cells is accompanied by a loss of CAV1 in the tumor stroma, both facts that were correlated with higher Gleason scores, poor prognosis, and pronounced resistance to therapy particularly to radiotherapy (RT). However, it needs to be clarified whether inhibiting the CAV1 gain in the malignant prostate epithelium or limiting the loss of stromal CAV1 would be the better choice for improving PCa therapy, particularly for improving the response to RT; or whether ideally both processes need to be targeted. Concerning the first assumption, we investigated the RT response of LNCaP PCa cells following overexpression of different CAV1 mutants. While CAV1 overexpression generally caused an increased epithelial-to-mesenchymal phenotype in respective LNCaP cells, effects that were accompanied by increasing levels of the 5′-AMP-activated protein kinase (AMPK), a master regulator of cellular homeostasis, only wildtype CAV1 was able to increase the three-dimensional growth of LNCaP spheroids, particularly following RT. Both effects could be limited by an additional treatment with the SRC inhibitor dasatinib, finally resulting in radiosensitization. Using co-cultured (CAV1-expressing) fibroblasts as an approximation to the *in vivo* situation of early PCa it could be revealed that RT itself caused an activated, more tumor-promoting phenotype of stromal fibroblats with an increased an increased metabolic potential, that could not be limited by combined dasatinib treatment. Thus, targeting fibroblasts and/or limiting fibroblast activation, potentially by limiting the loss of stromal CAV1 seems to be absolute for inhibiting the resistance-promoting CAV1-dependent signals of the tumor stroma.

## Introduction

Prostate cancer (PCa) is one of the most commonly diagnosed cancers worldwide with nearly 1.5 million new cases estimated for 2020 and nearly 400,000 burden related deaths (1, 2). Besides radical prostatectomy and radiotherapy (RT), androgen deprivation therapy has been established as common modality for patients with localized PCa (3). Unfortunately, depending on the grade of the PCa, most of the patients experience relapse due to incomplete tumor eradication or experience progression to castration-resistant PCa, formerly termed ‘hormone-refractory PCa’ (4–6). Today, there are innovative and effective treatment options for patients suffering from metastatic castration-resistant PCa, for example androgen receptor antagonists (e.g., abiraterone or enzalutamide, cytotoxic (taxane-based) chemotherapies (e.g., with docetaxel or cabazitaxel), radiopharmaceuticals (e.g., high-energy delivering Radium-223) or novel immunotherapies (3, 4, 6, 7). However, castration-resistant PCa remains incurable, with median survival times ranging from 9 to 30 months, and respectively from 9 to 13 months for metastatic castration-resistant PCa; and the prognosis remains really poor due to the development of primary resistances to these treatments as well as secondary resistances (3).

Acquired drug resistance by PCa cells has been closely linked to apoptosis evasion, a process that might be caused, at least in part, by stromal derived factors (8–10). Tumors modulate or more precisely ‘educate’ neighboring stromal cells to adopt a tumor-associated phenotype finally reinforcing tumor progression. Herein, the membrane protein caveolin 1 (CAV1) gained attraction because CAV1 expression levels strongly increase in malignant prostate epithelial cells at advanced tumor stages (11–13). In parallel, a loss of stromal, particularly fibroblastic CAV1 could be observed, which correlated with tumor progression and therapy resistance, and may therefore be suited as a prognostic marker. Particularly, activated fibroblasts, also termed cancer-associated fibroblasts (CAF), were shown to foster therapy resistance of malignant epithelial cells in a CAV1-dependent manner (12–15). Activated fibroblasts that were characterized by low CAV1 expression levels exhibited a more reactive tumor-promoting phenotype and induced resistance of PCa cells with differential CAV1 levels and of PCa cells with low endogenous CAV1 levels in a paracrine manner (16). Likewise, co-culturing CAV1-deficient PCa cells with CAV1-expressing fibroblasts resulted in an upregulation of CAV1 in respective PCa cells, strongly suggesting a transfer of CAV1 from CAV1-expressing fibroblasts (16). A re-distribution of CAV1 was further observed in malignant prostate epithelial cells upon progression of xenograft tumors grown from CAV1-deficient PCa cells, an effect that coincided with increased radiation resistance. Mechanistically, CAV1-deficient fibroblasts were shown to foster RT resistance of PCa cells by allocating CAV1-dependent apoptosis inhibiting proteins to the tumor cells, e.g., the p53-inducible cell survival factor TRIAP1 (TP53-regulated inhibitor of apoptosis 1, also known as p53CSV) (13).

However, it remains elusive whether the concurrently observed loss of stromal CAV1 is the cause and moreover prerequisite for the gain of CAV1 in malignant epithelial cells, or if epithelial CAV1 (re-)expressions in PCa cells upon disease progression induce the loss of stromal CAV1. Furthermore, it is still unclear, whether the loss of stromal CAV1 causes the observed gain in resistance to RT or rather is due to epithelial CAV1 upregulations, or a combination of both. Thus, it is important to dissect the characteristic stromal–epithelial CAV1 alterations within PCa, as a ‘simple’ CAV1-targeting would only achieve radiosensitization at the levels of tumor cells while a stromal CAV1 loss would counteract a desired therapeutic benefit. One potential CAV1-modulating target could be the non-receptor tyrosine kinase SRC. SRC-mediated CAV1 phosphorylation on tyrosine 14 (Tyr14) is supposed to be required for CAV1-dependent plasma membrane signaling, and also for the internalization of CAV1 from the plasma membrane (*via* the release of caveolae) (17, 18). SRC was already shown to significantly impact on PCa progression, particularly in castration-recurrent metastatic disease (19, 20). Furthermore, significantly higher SRC expression levels (together with higher CAV1 phosphorylation levels) were present in the malignant epithelial cells of advanced PCa tumors of higher Gleason grades, whereas immunoreactivities of both proteins were declined within the more reactive tumor stroma (16). Thus, SRC-dependent CAV1 signaling could be considered as integral part of PCa pathobiology and fundamentally impacts on RT resistance.

## Material and Methods

### Cell Cultures

The human PCa cell line LNCaP and the human skin fibroblast cell line HS5 were from ATCC (Manassas, VA) and cultured in RPMI Medium (Gibco, ThermoFisher, Waltham, MA) supplemented with 10% fetal bovine serum and 100 U/ml Penicillin/Streptomycin (Sigma-Aldrich, St. Louis, MO) under standard cell culture conditions (37°C, 5% CO2, 95% humidity). The cell lines were routinely tested for mycoplasma contamination (every two weeks) and periodic authenticated by STR profiling (if necessary, no later than yearly). LNCaP cells (with low endogenous CAV1 levels) were transduced with lentiviral SIN (self-inactivating) vectors, containing either the cDNA of wildtype CAV1 (CAV1 WT), the cDNA of CAV1 Y14F (substitution of Tyr by Phe on position 14) or the cDNA of CAV1 P132L (substitution of Pro to Leu on position 132) (21, 22). These vectors co-express enhanced GFP (green fluorescent protein) as a reporter gene. Lentiviral vectors were produced and titrated as previously described (23, 24). Within 2–5 days after transduction eGFP-positive cells were sorted in a FACS Vantage cell sorter (BD Biosciences, Heidelberg, Germany). For 3D spheroid cultures, indicated cells were cultured alone or in or in indicated combinations in normal growth medium (NGM) containing methylcellulose (ratio 1:2) as hanging drops for 24 h as previously described (25). Afterwards, spheroids were placed in growth factor-reduced Matrigel (Corning, NY) (diluted 1/2 with NGM). For inhibitor treatments, plated cells or spheroids were treated with 50 nM Dasatinib (BMS-354825), 100 nM Bosutinib (SKI-606), (drug stocks: 10 mM in DMSO; all from Selleckchem, Houston, TX) or vehicle control. Two hours later, cells were irradiated with indicated doses using an X-RAD 320 machine (Precision X-Ray Inc.) with 320 kV, 10 mA, and a 1.65 mm aluminum-filter at a distance of 50 cm (dose rate of 3.3 Gy/min) at room temperature. Pictures were taken directly and 48 h after treatment at ×10 magnification. Spheroid size was measured and calculated using ImageJ software (26). For the detection of cell death, spheroids were incubated thereafter for additional 15 min with 50 µg/ml propidium iodide and 1 µg/ml Hoechst 33342 (2’-[4-ethoxyphenyl]-5-[4-methyl-1-piperazinyl]-2,5’-bi-1H-benzimidazole trihydrochloride trihydrate; ThermoFisher, Waltham, MA) for nuclei staining and analyzed by phase contrast and fluorescent microscopy using an ZEISS Axio Observer (Carl Zeiss, Oberkochen, Germany) as previously described (25). Cellular proliferations were determined by crystal violet staining (0.1% crystal violet in PBS; Carl Roth, Karlsruhe, Germany) of PBS washed and glutaraldehyde-fixed cells. Following Triton X-100 dye release dye concentrations were measured spectrophotometrically at 540 nm. Cellular migrations were investigated *via* time lapse microscopy for the indicated time following radiation treatment as previously described (27). Briefly, cells were grown to confluence, irradiated and a thin wound was introduced by scratching with a 10 µl pipette tip. Wound closure was determined for the different treatments by measuring the migration distance using ImageJ. For clonogenic survival, cells were plated in six-well plates as previously described (25, 28). After additional 10 days of treatment Coomassie Brilliant Blue stained colonies (≥50 cells/colony) were counted and plating efficiencies (PE = number of colonies observed/number of cells plated) and survival curves were calculated (13, 25).

### Flow Cytometry

Cell cycle analysis, and reactive oxygen species (ROS) levels were analyzed at the indicated time points post RT using respectively harvested cells (by trypsinization) in combination with staining solution. For cell cycle analysis, Nicoletti staining solution [50 μg/ml propidium iodide (PI), 0.1% sodium citrate (w/v), and 0.05% Triton X-100 (v/v) in PBS] was used (30 min incubation at room temperature prior analyses). PI exclusion staining solution (10 µg PI/ml in PBS) was used for staining of death cells after 30 min incubations. ROS levels were evaluated by using 5 µM dihydroethidium (Sigma-Aldrich, St. Louis, MO) in PBS and 30 min incubation at 37°C. Mitochondrial ROS levels were determined by MitoSOX™ (ThermoFisher) at indicated time points post RT. Measurements were performed on a FACS Calibur flow cytometer (BD, Heidelberg, Germany).

### Western Blotting

Generation of whole cell lysates was carried out by scraping cells off into ice-cold RIPA buffer (150 mmol/L NaCl, 1% NP40, 0.5% sodium-deoxycholate, 0.1% sodium-dodecylsulfate, 50 mmol/L Tris/HCl, pH 8, 10 mmol/L NaF, 1 mmol/L Na3VO4) supplemented with Protease-Inhibitor cocktail (Roche). After 2–3 freeze and thaw cycles the protein content of the lysates was measured by using a Bio-Rad DC™ Protein Assay. Approximately 50–100 µg of total proteins were used for SDS-PAGE electrophoresis. Western blots were done as previously described (16, 24) and the indicated antibodies were used to detect protein expression levels. The CAV1 (D46G3; #3267), SRC (36D10; #2109) phosphorylated SRC Tyr527 (#2105), Tyr416 (E6G4R; #59548), vimentin (D21H3; #5741), AMPK (#2532), phosphorylated AMPK Thr172 (40H9; #2535), and phosphorylated HSP27 Ser82 (D1H2F6; #9709) antibodies were from Cell Signaling Technology (Danvers, MA; all 1:1000), against TAGLN from Proteintech (Chicago, IL), and beta-actin antibody (AC-74, A2228 was from Sigma-Aldrich). Representative blots from at least three independent experiments were shown.

### Large Lipid Platform (GM1) Staining

Lipid platforms were stained by using 0.1 mM AlexaFluor555-conjugated cholera-toxin B-subunit for 45 min as described before (25). Cells were fixed with 4% PFA for 10 min. After blocking with 10% bovine serum albumin (in PBS) for 20 min, co-staining with CAV1 was performed (dilution 1:100) in blocking buffer for 1 h. Nuclei were counterstained using 1 µg/ml Hoechst 33342 (in PBS) for 10–15 min after secondary antibody staining using goat anti-rabbit IgG H&L (Alexa Fluor^®^ 647; dilution 1:500 in blocking buffer). Samples were mounted with Fluoromount-G™ Mounting Medium (Thermo Fisher). Pictures were taken with a Leica confocal microscope of at least 10 (−20) cells per condition of each independent experiment.

### Extracellular Flux Analysis

Cells were plated at in XF96 microplates (Seahorse Bioscience, Agilent Technologies, Santa Clara, CA) according to the manufacturer’s instruction and as previously described (29). For Seahorse XF Cell Mito Stress Test, 24 h post treatment medium was changed to XF base medium, supplemented with 1 mM pyruvate, 2 mM glutamine and 10 mM glucose, and incubated for 1 h at 37°C in a CO_2_-free incubator. Mitochondrial oxidative phosphorylation on the basis of the oxygen consumption rate (OCR) and glycolysis by analyzing the extracellular acidification rate (ECAR) were estimated following oligomycin (LNCaP: 2 µM, HS5: 1 µM)), carbonyl-cyanide-p-trifluoromethoxyphenylhydrazone (FCCP) (LNCaP: 0.25 µM, HS5: 1 µM) and rotenone and antimycin A (both 0.5 µM) treatments at indicated time points using a Seahorse XFe 96 Analyzer. Hoechst 33342 (10 µg/ml, Thermo Fisher Scientific; Waltham, MA) was used for individual normalization to DNA.

### Cholesterol Quantification

Total cholesterol concentrations were determined by a coupled enzyme assay, which results in a colorimetric (570 nm) product proportional to the cholesterol present, from around 0.5 to 1 × 10^6^ LNCaP cells by HDL and LDL/VLDL Cholesterol Assay Kit (ab65390, Abcam, Cambridge, UK) according to the manufacturer’s instructions. Obtained cholesterol levels were related to respective protein concentrations (µg cholesterol per µg protein).

### Statistics

Unless otherwise indicated, data were obtained from at least 3 independent experiments (n = biological replicates). Data were presented as mean values ± SD or SEM. Data analyses were performed by indicated one- or two-way ANOVA followed by Tukey’s multiple comparison post-tests or by unpaired (two-tailed) t-tests using Prism7 (GraphPad, La Jolla, California). Statistical significance was set at the level of p ≤0.05.

## Results

### A Gain of Epithelial CAV1 Increases AMP-Activated Protein Kinase (AMPK)-Mediated Signaling as Well as the Concomitant Induction of an EMT Phenotype, Particularly in Combination With RT

**Figure 1:**
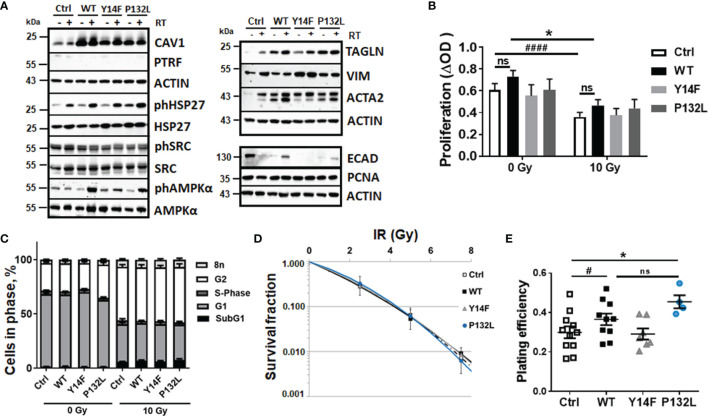
CAV1-dependent increases in AMP-activated protein kinase (AMPK)-mediated signaling accompanied by EMT phenotypes in LNCaP PCa cells. Expression levels of the indicated proteins were analyzed in whole protein lysates of cultured LNCaP PCa cells stably overexpressing the introduced CAV1 variants (wildtype CAV1, WT; phosphorylation-deficient CAV1, Y14F; the transmembrane localization affecting proline-132-to-leucine substituted CAV1, P132L) with or without radiation treatment (48 h after RT with 10 Gy) using Western blot analysis. Empty vector transduced LNCaP cells with low endogenous CAV1 levels served as controls [Ctrl (CAV-)]. **(A)** Representative blots from at least 4 independent experiments are shown. **(B)** Cell proliferation was determined using crystal violet staining. Data are summarized as mean values ± SEM of 6–13 independent experiments measured in quadruplets each. P-values indicate: *p ≤ 0.05 by one-way ANOVA with Tukey’s multiple comparison post-test and additionally by unpaired (two-tailed) t-tests depicted as ^####^p ≤ 0.001. **(C)** Cell cycle phases and apoptotic cells (subG1) were analyzed by flow cytometry. Graphs consist of data from 12–15 individual experiments (with SEM). The statistically significant differences within SubG1 (p ≤ 0.001), G1 (p ≤ 0.001), and G2 (p ≤ 0.001) values as estimated by two-way ANOVA with Tukey’s multiple comparison test (0 Gy versus 10 Gy for each CAV1 variant) were not depicted. **(D)** Clonogenic survival was evaluated 10 days post treatment. Coomassie stained colonies were quantified by counting. Data show the surviving fractions from at least 3 independent experiments (means ± SD) plated in triplicates each. **(E)** Plating efficiency was determined after 10 days following plating of low cell numbers (500 cells per 35 mm dish, plated in triplicates each). Symbols depict individual values from different independent experiments. P-value indicates: *p ≤ 0.05 by one-way ANOVA with Tukey’s multiple comparison post-test and additionally by unpaired (two-tailed) t-test depicted as ^#^p ≤0.05. ns, not significant.

We previously reported that reducing CAV1 expression levels in advanced PC3 PCa cells re-sensitized the cells to RT (16). In contrast, the associated loss of CAV1 in stromal fibroblasts mediated an activated, more reactive fibroblast phenotype that fostered RT resistance of adjacent malignant prostate epithelial cells by shuttling apoptosis inhibiting proteins (13, 16). However, it is not yet clear whether the loss of stromal CAV1 or the gain of epithelial CAV1 precedes and/or induces the characteristic stromal–epithelial CAV1 substitutions, and which of the two facts is more decisive for RT failure. In order to gain insight into the latter fact, LNCaP PCa cells with low endogenous CAV1 expression levels were transduced with different CAV1 mutants and respective cultures stably overexpressing the introduced CAV1 variants were generated (**Figure 1**). Besides wildtype CAV1 (CAV1 WT), the phosphorylation-deficient CAV1 Y14F mutant was overexpressed as well as the CAV1 P132L mutant known to ‘mis-localize’ CAV1 away from the plasma membrane. Empty vector transduced LNCaP cells served as controls [Ctrl (CAV−)].

Increased cellular CAV1 expression levels were first analyzed on protein levels by Western blot analyses (**Figure 1A**). Ectopic expressions of the indicated CAV1 variants efficiently generated LNCaP cells cultures stably overexpressing CAV1 (**Figure 1A** and **Supplementary Figure S1**). No potentially induced expression of cavin-1, also known as polymerase I and transcript release factor (PTRF), as CAV1 interaction partner necessary for the caveolae-assembly could be detected. Further examinations of the expression levels of the survival protein heat shock protein 27 (HSP27), a multidimensional protein which acts as a protein chaperone, revealed increased levels of phosphorylated HSP27 in response to RT. No obvious differences could be estimated upon induced CAV1 expressions, although phosphorylated HSP27 levels showed a slight trend to be increased in CAV1 WT and CAV1 P132L-expressing LNCaP cells (**Figure 1A** and **Supplementary Figure S1**). Although respective phosphorylation levels were not affected, associated AMP-activated protein kinase (AMPK) expression levels acting as metabolic sensors were found to be increased in CAV1-expressing LNCaP PCa cells, particularly in CAV1 Y14F and CAV1 P132L cells, with slightly but not significantly increased levels of AMPK following RT. Respective SRC expression levels and SRC activities as indicated by SRC phosphorylation, were not affected upon expression of different CAV1 mutants in LNCaP cells. Of note, the gain in CAV1 expressions caused an upregulation of the epithelial-to-mesenchymal (EMT) markers smooth muscle actin (ACTA2) and transgelin (TAGLN), a transforming growth factor beta (TGFβ)-inducible protein, in respective cultures. Both markers showed even increased expression levels following RT in Ctrl LNCaP cell cultures (**Figure 1A** and **Supplementary Figure S1**). No obvious differences could be detected for the EMT marker protein vimentin. In addition, significant reductions of epithelial cadherin expression levels became prominent following increased CAV1 expressions. The proliferation marker proliferating-cell-nuclear-antigen was either not affected upon increased CAV1 expressions in all cells, neither following RT treatment. We then investigated the effects of increased CAV1 expression levels on functional cellular features. No obvious impact on cellular proliferation levels could be observed with slight (but not significant) increases estimated in CAV1 WT and CAV1 P132L LNCaP cultures, even after RT (**Figure 1B**). CAV1 Y14F-expressing LNCaP PCa cells showed rather proliferation rates comparable to Ctrl (CAV1−) LNCaP cells with a low endogenous CAV1 content. No differences within the cell cycle distributions were observed following increased CAV1 expressions (**Figure 1C**). RT caused increased cell numbers of cells being in G2 phase. Decreasing cell numbers in G1 phase were accompanied by increased subG1 fractions, indicating apoptotic cells. However, no differences concerning the introduced CAV1 mutants were detected. The low levels of apoptotic cells (around 5%) 48 h post RT with 10 Gy further stressed the quite radioresistant nature of the used LNCaP cells. Accordingly, total cells death levels using a propidium iodide exclusion assay only revealed cell death levels of 2–6% following RT, with slightly decreased cell death levels of LNCaP cells that overexpressed different CAV1 mutants (not shown). Even within long-term assays measuring the surviving fraction after RT it was revealed that the number of epithelial LNCaP cells able to re-grow and form a colony after irradiation were not obviously affected by the increased CAV1 levels (**Figure 1D**). We attributed that to the fact that LNCaP cells *per se* were already quite radioresistant, and thus the gain of additional CAV1 did not reveal further differences at RT doses <7.5 Gy. Of note, the plating efficiencies of LNCaP CAV1 WT cells and also of LNCaP CAV1 P132L were increased compared to Ctrl (CAV1−) and CAV1 Y14F LNCaP cells (**Figure 1E**).

**Figure 2:**
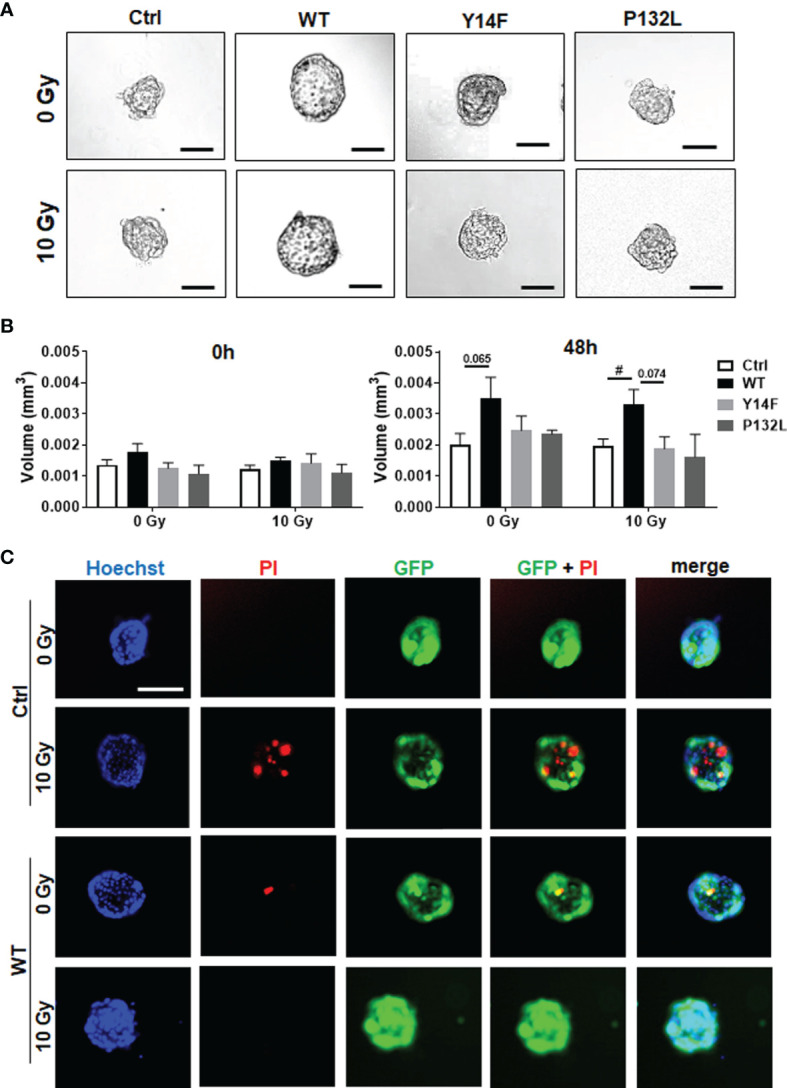
CAV1 WT-expressing LNCaP PCa cells exhibit an increased spheroid growth while lacking RT-induced growth retardation. LNCaP PCa cells stably overexpressing the introduced CAV1 variants (wildtype CAV1, WT; phosphorylation-deficient CAV1, Y14F; proline-132-to-leucine substituted CAV1, P132L) as well as control (Ctrl) LNCaP cells were cultured in hanging drops for 24 h. After formation of spheroids, cells were plated in growth factor-reduced Matrigel mixed with normal growth medium (1/2, v/v) and irradiated with 0 Gy or 10 Gy. **(A)** Representative phase contrast images 48 h post radiation treatment (0 and 10 Gy) are shown. Scale bar represents 150 µm. **(B)** Spheroid growth was measured at the time of irradiation (0 h) and 48 h later and respective volumes were calculated. Graphs depict the measurements from 4 to 8 independent experiments where at least 10 spheroids per condition were measured. P-value indicates: ^#^p ≤ 0.05 by unpaired (two-tailed) t-test. **(C)** Cell death was analyzed afterwards by fluorescence microscopy using propidium iodide. Hoechst 33342 was used for nuclei staining. Representative fluorescent photographs from CAV1 WT and Ctrl LNCaP cells (with low endogenous CAV1 levels), both expressing the reporter green fluorescent protein (GFP), are exemplarily shown (48-hour time point). Scale bar represents 100 µm.

**Figure 3:**
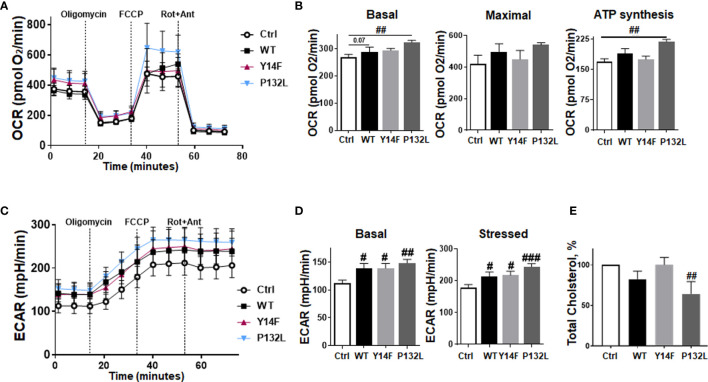
Induced CAV1 expressions do not obviously affect mitochondrial respiration rates in LNCaP PCa cells but increase respective glycolysis levels. Extracellular flux analyses were performed in order to measure oxygen consumption rates (OCR) and extracellular acidification rates (ECAR) in CAV1 WT, CAV1 Y14F, CAV1 P132L and also in control (Ctrl) LNCaP PCa cells over time, while oligomycin (Oligo; 2 µM), FCCP (0.25 µM) and rotenone/Antimycin A (Rot/Ant; 0.5 µM) were added at indicated time points. **(A)** OCR levels are shown. **(B)** Basal respiration, maximal respiration, and ATP production were depicted in separate bar diagrams. **(C)** Respective ECAR measurements over time are shown. **(D)** Basal and stressed (following oligomycin treatment) measurements of ECAR were depicted in separate bar diagrams. Data were summarized as mean values ± SD (measured in 4–7 replicates each). One of 3–4 independent experiments with similar results is exemplarily shown. P-values indicate: ^#^p ≤ 0.05, ^##^p ≤ 0.01, ^###^p ≤ 0.005 by unpaired (two-tailed) t-tests. **(E)** Total cholesterol concentrations were determined by a coupled enzyme assay, which results in a colorimetric (570 nm) product proportional to the cholesterol present. Total cholesterol levels of Ctrl (CAV1−) LNCaP cells were set as 100%. Data are shown as means ± SEM of different biological replicates (Ctrl (CAV1−): n = 7; CAV1 WT: n = 10; CAV1 Y14F: n = 6; CAV1 P132L: n = 3. P-value indicates ^##^p ≤ 0.01 by unpaired (two-tailed) t-test (versus Ctrl).

We then investigated CAV1-dependent cell growth and invasion in a more approximated *in vivo* situation using 3D cultures with spheroids embedded in growth-factor-reduced Matrigel (**Figure 2**). At least by tendency, an increased spheroid growth was detected for CAV1 WT LNCaP cells indicating an increased invasion potential, an effect that became significant following RT (**Figures 2A, B**). The growth and RT response of CAV1 Y14F LNCaP spheroids resembled more that of Ctrl (CAV1−) LNCaP·cells, indicating that phosphorylation-deficient CAV1 mimics the effect of CAV1-deficiency, corroborating its action in a dominant-negative manner. CAV1 P132L LNCaP spheroids were not increased in size and showed rather smaller volumes after RT. After RT, the presence of tumor cells that were permeable for propidium iodide, and thus clearly showed RT-­induced cell death, become prominent in LNCaP spheroids, an effect that was less pronounced in CAV1 WT LNCaP cells (**Figure 2C**). However, no impact on the migration potential could be estimated in LNCaP cells following increased CAV1 expression levels under non-irradiated conditions, while CAV1 WT expressing LNCaP cells showed an increased migration capacity following RT (**Supplementary Figure S2**). As tendentially increased proliferation rates together with the increased three-dimensional spheroid growth were detected in CAV1 WT LNCaP PCa cultures, the impact of CAV1 in metabolic reprogramming was next analyzed by extracellular flux measurements (**Figure 3**). Mitochondrial function was analyzed in LNCaP cultures stably expressing the different CAV mutants upon oligomycin-mediated ATP synthesis inhibition, upon FCCP-uncoupled oxidative phosphorylation, and upon blocking mitochondrial respiration with rotenone and antimycin (**Figure 3A**). Whereas CAV1 WT LNCaP cells exhibited similar mitochondrial respiration rates like Ctrl (CAV1−) and CAV1 Y14F LNCaP cells, CAV1 P132L cells showed increased mitochondrial respirations with increased basal and maximal respiration levels, and thus increased rates of adenosine triphosphate (ATP) production (**Figures 3A, B**). Of note, gains in cellular CAV1 were accompanied by increases in glycolysis levels as measured through increases of the extracellular acidification rates (ECAR) of the surrounding media, that results from the excretion of lactic acid after its conversion from pyruvate (**Figure 3C**). Besides the increase in basal glycolysis levels following increased cellular CAV1 levels, additional increases of glycolysis in response to metabolic stress (upon ATP synthesis inhibition by oligomycin) could be detected indicating gains in the metabolic potentials (**Figures 3C, D**). CAV1 P132L cells showed the strongest impact. However, cellular ROS levels were not affected following the constitutive expression of CAV1 and also of both CAV1 mutants, nor following RT (**Supplementary Figure S3A**). As CAV1 is an important regulator of cholesterol homeostasis, and cancer cells—besides its decisive impact on membrane physical properties—utilize cholesterol to satisfy their increased nutrient demands, the cholesterol contents of the different CAV1 variants were determined (**Figure 3E** and **Supplementary Figure S3B**). Of note, a significantly reduced cholesterol content was detected for CAV1 P132L LNCaP cells, while CAV1 WT and CAV1 Y14F LNCaP cells showed similar cellular cholesterol levels compared to Ctrl (CAV1−) cells. The CAV1-dependent and cholesterol-mediated modifications of the plasma membrane could affect biophysical properties and coalesce signaling-related biomolecules into specialized microdomains, finally fostering PCa cancer cell proliferation, invasion and survival. Therefore, the presence of (large) lipid platforms (LLP) being highly enriched in ganglioside M1 (GM1) was additionally analyzed (**Supplementary Figure S3C**). According to the reduced cholesterol content of CAV1 P132L LNCaP cells, reduced levels of GM1-containing signaling platforms were estimated here, while CAV1 WT and CAV1 Y14F LNCaP cells showed LLP levels compared to Ctrl (CAV1−) cells. These results suggest that the increase in the metabolic demands of non-plasma membrane targeted CAV1 P132L in LNCaP cells may compensate reduced cholesterol utilization due to decreased cholesterol levels.

### SRC Inhibition by Dasatinib Treatment Acts as Radiosensitizer in LNCaP Cells With Differential CAV1 Levels and Limits PCa Cell Invasion

**Figure 4:**
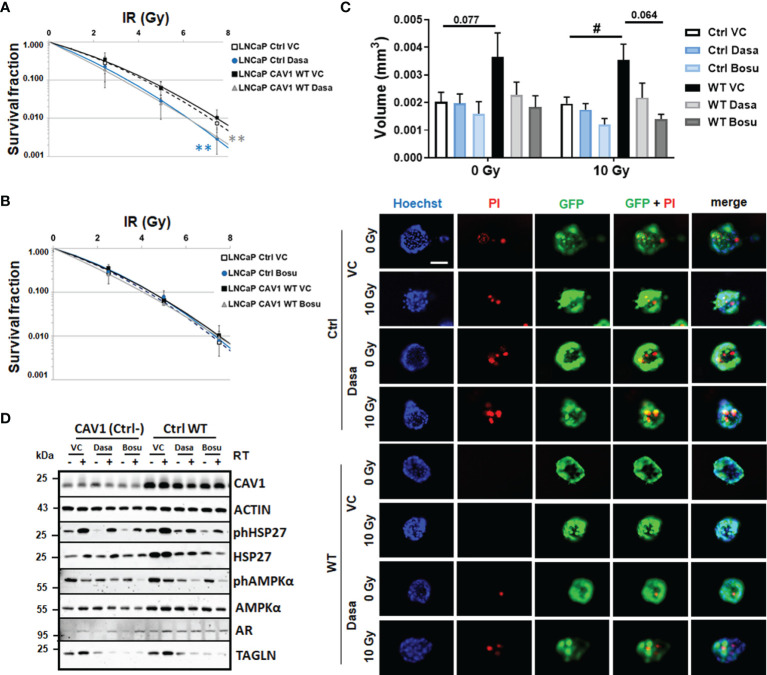
SRC inhibition by dasatinib efficiently reduces the clonogenic survival of LNCaP cells with differential CAV1 levels and also reduces three-dimensional PCa cell growth. LNCaP PCa cells stably overexpressing the introduced WT CAV1 and control (Ctrl) LNCaP cells with low endogenous CAV1 levels were cultured in normal growth media supplemented with the SRC inhibitors dasatinib, bosutinib or vehicle control (VC) 2 h prior radiation treatment with the indicated doses (0–7.5 Gy). Clonogenic survivals were evaluated 10 days post treatment with dasatinib **(A)** or bosutinib (**B**). Coomassie stained colonies were quantified by counting. Data show the surviving fractions from at least 3 independent experiments (means ± SD) plated in triplicates each. P-values indicate: **p ≤ 0.01 by one-way ANOVA with Tukey’s multiple comparison post-test. **(C)** Growth patterns of CAV1 WT and control LNCaP spheroids were evaluated 48 h post RT (10 Gy) and combined dasatinib treatment. Graphs depict the measurements from 4 to 6 independent experiments where at least 10 spheroids per condition (and per experiment) were measured. P-value indicates: ^#^p ≤ 0.05 by unpaired (two-tailed) t-test. Cell death was analyzed afterwards by fluorescence microscopy using propidium iodide. Hoechst 33342 was used for nuclei staining. Representative fluorescent photographs from CAV1 WT and Ctrl LNCaP cells (with low endogenous CAV1 levels) are shown (48-hour time point). Scale bar represents 100 µm. **(D)** Expression levels of the indicated proteins were analyzed in whole protein lysates of cultured CAV1 WT and control LNCaP PCa with or without radiation and SRC inhibitor (dasatinib and bosutinib) treatment (48 h after RT with 10 Gy) using Western blot analysis. Representative blots at least 3–4 independent experiments are shown.

CAV1-dependent plasma membrane signaling depends—among others—on CAV1 tyrosine-14 phosphorylation mediated by non-receptor SRC-family tyrosine kinases. Increased SRC levels or particularly increased SRC activity could decisively foster advanced cellular features that were previously seen in malignant epithelial cells upon PCa progression (16). SRC further on was shown to mediate tyrosine phosphorylation of androgen receptor and in turn androgen-stimulated androgen receptor-mediated HSP27 phosphorylation, levels which were found to be increased following RT. We therefore investigated whether SRC-inhibition could be used as potential strategy to inhibit CAV1-dependent increases in invasion and migration and if SRC inhibition could potentially function as radiosensitizing strategy. Therefore, LNCaP cultures either overexpressing CAV1 WT or control cells with a low endogenous CAV1 content were treated with the SRC inhibitors dasatinib or bosutinib alone or in combination with RT (**Figure 4**). While both treatments only minimally reduced respective proliferation rates either without or with combined RT (not shown), dasatinib treatment efficiently sensitized both LNCaP variants to RT as estimated by significantly reduced clonogenic survival rates (**Figure 4A**). However, no radiosensitization was achieved by bosutinib (**Figure 4B**), although bosutinib, similarly to dasatinib, efficiently reduced the increased plating efficiency of CAV1-expressing LNCaP PCa (**Supplementary Figure S4**). Similarly, the growth of 3D-cultured spheroids was reduced upon dasatinib and bosutinib treatment even when combined with RT, an effect that was more prominent for CAV1 WT LNCaP spheroids that showed an increased spheroid growth (**Figure 4C**). Of note, treatments with both SRC inhibitors increased propidium iodide-positive cells within the respective cultures, even without RT co-treatment, indicating increased cell death levels upon treatment (**Figure 4C**). Western blot analyses further revealed that SRC-inhibition tends to reduce respective CAV1 levels in CAV1 WT LNCaP cells, an effect that was not significant 48 h after treatment (**Figure 4D** and **Supplementary Figure S5**). Although SRC inhibitor treatment did not impact on HSP27 phosphorylation levels, total HSP27 levels were found to be decreased in CAV1 WT expressing LNCaP and Ctrl cells. Likewise, levels of AMPK were reduced. The increased expression levels of the EMT marker TAGLN following RT were only tendentially decreased 48 h after dasatinib or bosutinib treatment, indicating that the CAV1-induced EMT phenotype in respective LNCaP cultures could be reduced, presumably upon prolonged treatment (**Figure 4D** and **Supplementary Figure S5**).

Conclusively, a gain of CAV1 in malignant prostate epithelial cells was associated with a more mesenchymal and thus more invasive phenotype that could even account for an increase in radioresistance. The use of SRC inhibitors, namely dasatinib and bosutinib efficiently reduced these phenotypes and increased the radiosensitivity of CAV1 expressing LNCaP cells. Furthermore, CAV1 localization and function seemed to be important for the respective phenotypes. Whereas the phosphorylation-deficient CAV1 expressing LNCaP cells seemed to mimic more the effects of CAV1-deficiency, LNCaP cells expressing miss-localized CAV1, due to the CAV1 P132L mutation, showed a similar increased mesenchymal phenotype while lacking increases in the invasion potential. In contrast, CAV1 P132L LNCaP cells showed a more radiosensitive phenotype, or specifically showed no reduced RT response, while exhibiting higher metabolic demands, both aspects that could be based on membrane alterations caused by the reduced cellular cholesterol content.

### RT Increases the Metabolic Potential of Stromal Fibroblasts, An Effect That Limits the RT Response of LNCaP PCa Cells in Spheroid Co-Cultures

**Figure 5:**
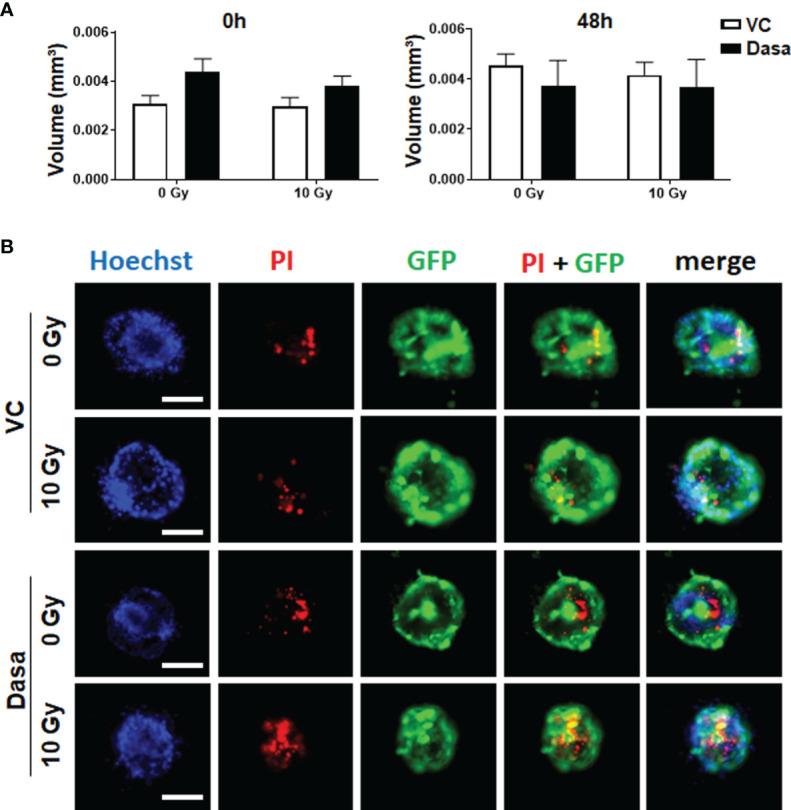
Stromal fibroblasts limit the radiosensitizing effect of SRC inhibition by dasatinib in LNCaP spheroid co-cultures. CAV1 expressing stromal fibroblasts (HS5) were co-cultured with LNCaP control (Ctrl) cells expressing low endogenous CAV1 levels and the reporter green fluorescent protein (GFP) in hanging drops for 24 h. After formation of spheroids, cells were plated in growth factor-reduced Matrigel mixed with normal growth medium (1/2, v/v) supplemented with dasatinib or vehicle control (VC) and irradiated with 0 Gy or 10 Gy. Pictures were taken at the time of irradiation (0 h) and 48 h later. **(A)** Spheroid growth was measured and the respective volumes were calculated for 0 and 48 h after irradiation. Data were derived from 3 to 5 individual experiments, where at least 10 spheroids per condition and per experiment each were measured. **(B)** Cell death was analyzed afterwards by fluorescence microscopy using propidium iodide. Hoechst 33342 was used for nuclei staining. Representative fluorescent images from the individual experiments are shown (48 h time point). Scale bars represent 100 µm.

**Figure 6:**
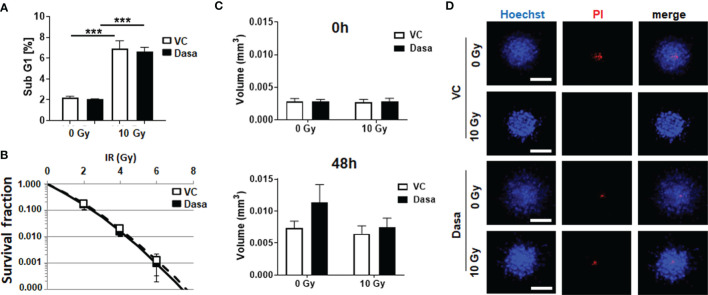
Dasatinib treatment does not impact on survival or growth of stromal fibroblasts, nor in combination with RT. CAV1 expressing stromal fibroblasts (HS5) were cultured as monolayers **(A, B)** or spheroids **(C, D)** with or without radiation treatment (0 and 10 Gy) in the presence of dasatinib or vehicle control (VC), **(A)** Apoptotic cells (subG1) were analyzed 48 h post treatment by flow cytometry. Graphs consist of data from 3 individual experiments (with SEM). P-values indicate ***p ≤0.005 as estimated by one-way ANOVA with Tukey’s multiple comparison test. **(B)** Clonogenic survival was evaluated 10 days post treatment by counting respective colonies. Data show the surviving fractions from 3 independent experiments (means ± SD) plated in triplicates each. **(C)** Spheroid growth was measured and the respective volumes were calculated for 0 and 48 h after irradiation from 3 to 4 individual experiments where at least 10 spheroids per condition and per experiment each were measured. **(D)** Cell death was analyzed afterwards by fluorescence microscopy using propidium iodide. Hoechst 33342 was used for nuclei staining. Representative fluorescent images from individual experiments are shown (48 h time point). Scale bar represents 100 µm.

**Figure 7:**
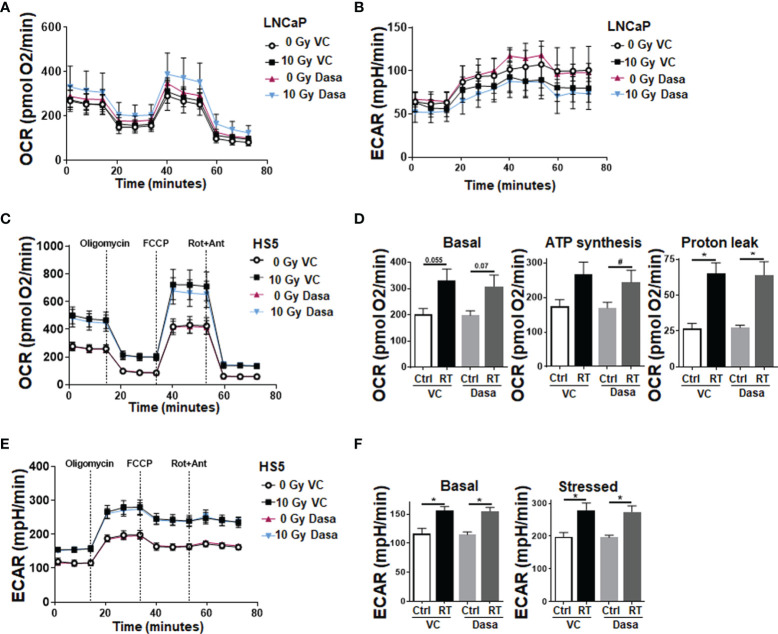
RT treatment causes an increased metabolic potential of fibroblasts, an effect that is not affected upon SRC inhibition; LNCaP PCa cells are not affected. Extracellular flux analyses of LNCaP and fibroblast cell cultures were performed following RT treatment (0 and 10 Gy) in the presence of dasatinib or vehicle control (VC) 24 h post treatments. OCR **(A)** and ECAR **(B)** levels of LNCaP PCa cells are shown. Data were summarized as mean values ± SD (measured in 4–7 replicates each). **(C)** OCR levels of stromal HS5 fibroblasts are shown. **(D)** Basal respiration, ATP production and proton leak were depicted in separate bar diagrams. **(E)** Respective ECAR measurement over time are shown. **(F)** Basal and stressed (following oligomycin treatment) measurements of ECAR were depicted in separate bar diagrams. Data were summarized as mean values ± SD (measured in 4–7 replicates each). P-values indicate: *p ≤ 0.05, by one-way ANOVA with Tukey’s multiple comparison test and additionally by unpaired (two-tailed) t-tests depicted as ^#^p ≤0.05.

With respect to the complex *in vivo* situation, it is not clear whether the gain in epithelial CAV1 or the loss of stromal CAV1 might be decisive for improving the therapy response of advanced PCa, particularly to RT. As SRC inhibition is suggested to additionally stabilize stromal CAV1 thereby limiting the gain in stromal radioresistance, spheroids consisting of normal LNCaP PCa cells with low endogenous CAV1 levels and of CAV1-expressing HS5 fibroblasts (to mimic the human situation concerning the epithelial-stromal CAV1 distributions at early stage PCa) were treated with RT in the presence or absence of SRC inhibition by dasatinib (**Figure 5**). Co-cultures spheroids containing HS5 fibroblasts showed an increased spheroid growth compared to LNCaP cells alone (**Figure 5A** and **Supplementary Figure S6**). Of note, dasatinib treatment did not impact on LNCaP spheroids containing CAV1-proficient HS5 fibroblasts (**Figure 5A**). Similarly, no additional (and expected radiosensitizing) effects were observed following RT, at least not concerning growth retardation at the 48 h time-point following post RT. In contrast increasing cell death levels could be estimated in these spheroids following dasatinib treatment as estimated by the increased positivity for propidium iodide (**Figure 5B**). Co-localization of propidium iodide-positivity and green fluorescent protein (GFP) expressing LNCaP cells within respective spheroids might point towards the fact that LNCaP cells were predominantly subjected to cell death following RT (**Figure 5B**). A similar response was estimated for another PCa cell line, namely 22Rv1 (androgen responsive) cells upon co-culture with CAV1-expressing fibroblasts, to exclude LNCaP-specific effects (**Supplementary Figure S7**). Again, increasing cell death levels could be estimated in these spheroids following dasatinib treatment. For HS5 fibroblasts cultured alone in turn, SRC inhibition did not reveal any harmful effects because apoptosis levels and also the long-term survival following RT either alone or in combination with dasatinib were not affected (**Figures 6A, B**). SRC inhibition in CAV1-expressing fibroblasts rather seemed to be beneficial, as a slightly improved spheroid growth was detected upon dasatinib treatment, which caused no obvious growth retardation following RT (**Figure 6C**). Accordingly, only single propidium iodide-positive cells were detected in these spheroids even after RT (**Figure 6D**). RT was already shown to foster a more reactive CAF-like phenotype in CAV1-expressing fibroblasts (13, 16). However, concerning the signaling as investigated above for respective LNCaP cell cultures, no alterations could be estimated upon dasatinib treatment (**Supplementary Figure S8**). We therefore investigated potential metabolic alterations following RT and combined dasatinib treatment in both cell types, namely LNCaP cells with low endogenous CAV1 levels and CAV1-expressing fibroblasts, resembling the initial situation of CAV1 distribution levels within early PCa (**Figure 7**). Whereas RT, either alone or in combination with dasatinib treatment, did not significantly impact the metabolic potential of LNCaP cells (**Figures 7A, B** and **Supplementary Figure S9A**), RT caused increased levels of mitochondrial oxygen consumption as shown by significantly increased basal and maximal respiration levels and increased levels of oxygen-linked ATP in CAV1-expressing HS5 fibroblasts (**Figures 7C, D**). Mitochondria inefficiency as indicated by an increased proton leak following RT in normal fibroblasts, was accompanied by an increase in glycolysis as determined through increased ECAR levels of the surrounding media (**Figures 7E, F**). Upon inhibition of ATP synthesis by oligomycin, non-irradiated as well as RT-treated fibroblasts cells showed a further slight increase in glycolysis (**Figures 7E, F**). Dasatinib did not further affect these alterations, and neither exhibited an effect when used without a combined RT treatment. Thus, RT induced phenotypic and metabolic alterations in stromal fibroblasts caused an activated fibroblast phenotype that was associated with an increased metabolic potential enabling adjacent tumor cell support, effects that were not affected, or particularly limited upon SRC inhibition (**Supplementary Figures S9B, C**).

Conclusively, SRC inhibition efficiently improved the RT response of LNCaP cells either being CAV1 pro- or deficient, while there is a lack of an improved RT response in the co-cultures of LNCaP PCa cells with stromal fibroblasts. Therefore, we conclude that, the loss of stromal CAV1, or more precisely the activation of stromal fibroblasts either by co-cultured PCa cells, by RT itself or presumably by a combination of both outweighs a potential gain of epithelial CAV1 and thus would be more decisive for RT therapy failure. The increased metabolic potential of stromal fibroblasts compared to PCa (**Supplementary Figure S9D**) cells further suggests that targeting fibroblasts and/or limiting fibroblast activation and induction of a CAF-like phenotype are absolute to inhibit the resistance-promoting CAV1-dependent signals of the tumor stroma. Particularly, limiting the loss of stromal CAV1 that can be observed following PCa progression could limit the stromal support of adjacent PCa cells (**Supplementary Figures S9E, F**).

## Discussion

### The Impact of a Proper CAV Localization and Function for the RT Response of Malignant Prostate Epithelial Cells

The lack of CAV1 in epithelial cells seems to be necessary for the epithelial phenotype *per se*, as it was shown to account for increased expressions of epithelial, but reduced expressions of EMT genes in PCa cells. At advanced tumor stages however, the gain in epithelial CAV1 was associated with increased invasion and metastasis and also therapy resistance, particularly to RT. Here we used LNCaP PCa cells stably expressing either wildtype CAV1, the phosphorylation-deficient CAV1 Y14F mutant or the CAV1 P132L mutant known to ‘mis-localize’ CAV1 away from the plasma membrane in order to investigate how a proper CAV1 localization and functionality impacts on the RT response of malignant prostate epithelial cells. Whereas CAV1 overexpression of all mutants increased the mesenchymal phenotype of respective cells, as revealed by the increased expression levels of mesenchymal proteins including TAGLN, only the introduced WT CAV1 gene caused a more invasive phenotype and concurrently a more radioresistant phenotype. CAV1 was particularly shown to induce a mesenchymal-like phenotype in castration-resistant PCa cells (30). Likewise, the reduction of CAV1 in CAV1-expressing PCa cells increased the expression of epithelial proteins finally favoring epithelial integrity and inhibiting invasion (31). We already suggested that a CAV1-dependent EMT in PCa progression may account for the observed resistance to RT, an effect that was accompanied by increased TAGLN immunoreactivities in human advanced PCa specimen (13, 16). As an actin-monomer binding protein, TAGLN was even found to be overexpressed in different cancer entities, and correlated with advanced prognostic features (32–34). Within the malignant epithelial cells, TAGLN promoted clonogenicity and increased cell migration and also invasion by inducing invadopodia formation and EMT. This led to the suggestion that actin-binding proteins could serve as potential biomarkers in cancer diagnosis and therapy, as these molecules significantly impact on cancer cell invasion and metastasis based on their actin-modulating action (35).

For PCa invasion particularly, CAV1 phosphorylation seems to be necessary, as phosphorylated CAV1, functioning in scaffolds, promoted PCa cell migration, an effect that was sensitive to SRC inhibition (36). Mechanistically, SRC was shown to mediate CAV1 phosphorylation on tyrosine-14 (Tyr14) following oxidative stress including RT, and growth factors stimulations. The other way around, phosphorylated CAV1 fostered the recruitment of SRC to the plasma membrane. Accordingly, orthotopic PCa-derived tumors exhibited increased SRC expression levels, an effect that was associated with CAV1 (re)expressions in malignant prostate epithelial cells and resistance to RT (16). Increased levels of phosphorylated CAV1 and SRC were even found within malignant epithelial cells of advanced human prostate adenocarcinomas (16). Therefore, it is reasoned that SRC-dependent CAV1 signaling decisively contributes to PCa progression and therapy resistance. Especially the metastatic spread of PCa was shown to depend on a gain in epithelial CAV1, and SRC-family kinase inhibitor treatments including PP2 and dasatinib efficiently reduced the metastatic features together with an accompanied reduction in CAV1 Tyr14 phosphorylation (37). In contrast, we did not detect significant alterations in SRC activities upon introducing wildtype CAV1 or CAV1 mutants in LNCaP cells, suggesting that CAV1-mediated signaling, at least in LNCaP PCa cells, might depend on other signaling pathways and not preferring SRC signaling.

**Figure 8:**
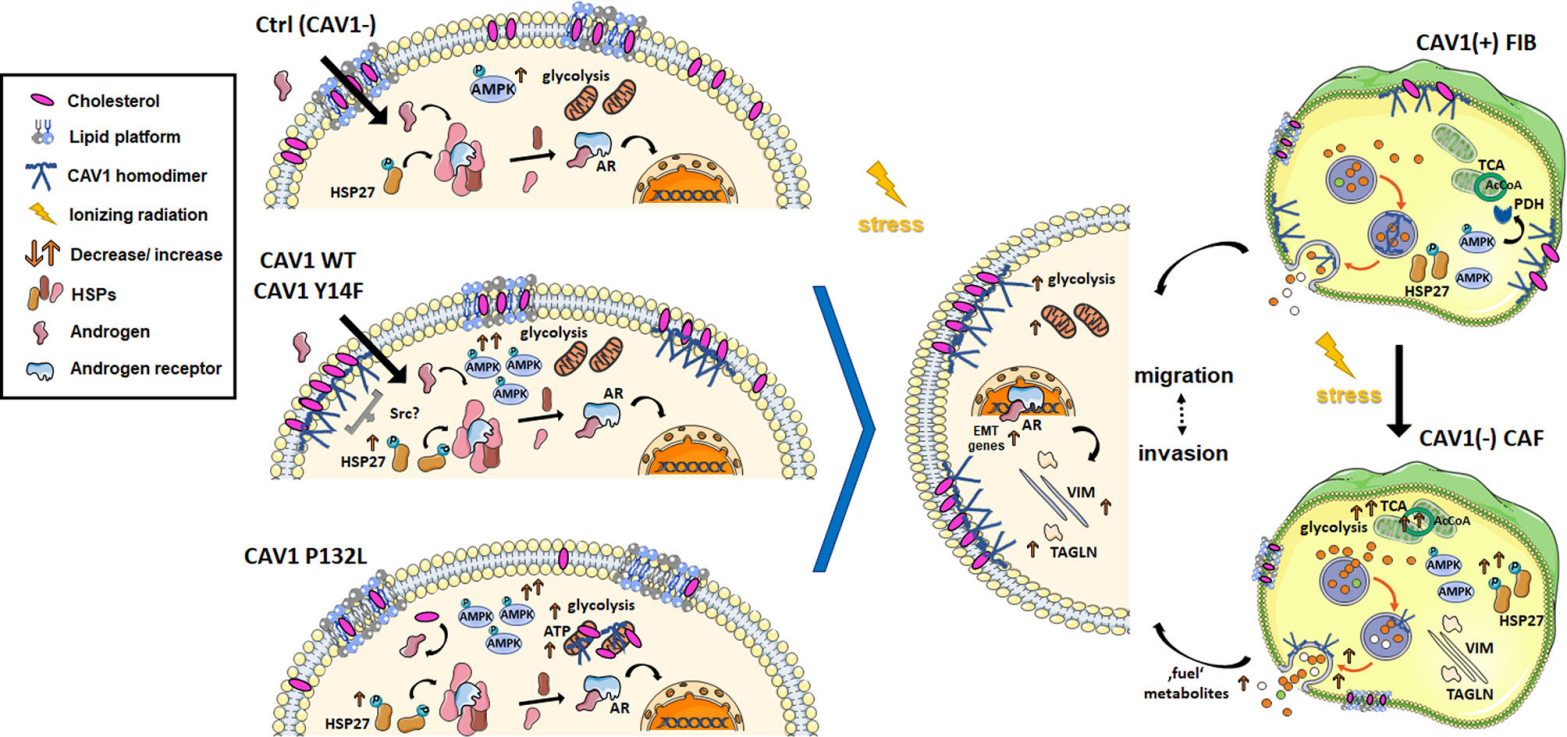
RT-induced stromal alterations decisively impact on adjacent prostate carcinoma (PCa) cells. PCa progression is associated with a critical shift of epithelial–stromal CAV1 expression levels. A re-expression of CAV1 within the malignant epithelial cells could be associated with epithelial-to-mesenchymal transition (EMT), an increased three-dimensional spheroid growth and RT resistance as investigated here. Concerning the related signaling, CAV1 seems to are promote adenosine monophosphate-activated protein kinase (AMPK) and potentially heat shock protein 27 (HSP27) activities, at least in combination with RT. As an assumption, increased HSP27 phosphorylations could foster (androgen-independent) androgen receptor (AR) signaling by displacing other HSPs (e.g., HSP90) from the cytosolic complex, with the AR subsequently squiring into the nucleus modulating target gene (e.g., EMT gene) expressions. It was further shown here, that PTRF (cavin-1)-deficient LNCaP PCa cells exhibit long-lasting high-density GM1 ganglioside containing signaling domains that were not affected following a gain in CAV1. In general, upon CAV1 re-expressions, either following increased cellular stress (e.g., by radiation treatment, RT) or by ectopic expression of CAV1, CAV1 can be found at the plasma membrane in non-caveolae plasma membrane domains, so-called scaffolds. Accompanied cholesterol binding reduces the amount of available cholesterol and thus the precursor for the synthesis of the steroid hormones. We therefore speculate that CAV1-dependent receptor signaling and raft-dependent endocytosis might account for signalosome-amplified signaling events that finally foster PCa cells growth, invasion and therapy resistance. Herein CAV1 Tyr14 phosphorylation seemed not to be required for the increased invasion potential of CAV1-expressing LNCaP cells, as it was shown within the present study that CAV1 Y14F-expressing LNCaP cells showed a similar EMT-phenotype but lacked increased three-dimensional spheroid growth. Similarly, non-plasma membrane-localized CAV1 was associated with an EMT phenotype while lacking increased spheroid growth. A gain of epithelial CAV1 could further be linked to increasing metabolic demands that were managed by increased glycolysis levels. Within CAV1 P132L-expressing LNCaP PCa cells, the strongest increase in glycolysis could be observed that, together with increased mitochondrial respiration rates, may compensate a reduced cholesterol utilization due to decreased cholesterol levels. RT-induced HSP27 signaling together with an increased AMPK-dependent signaling might even foster other metabolic processes to generate energy and biomolecules, e.g., fatty acid oxidation. In contrast, an activated fibroblast phenotype either following RT or potentially following ‘education’ by adjacent cancer cells will act tumor supporting, which in turn limits applied therapies. Likewise, a loss of stromal CAV1 was already shown to come along with a more reactive fibroblast phenotype as well as increased radioresistance of advanced PCa. Particularly increases in the metabolic potential as shown here following RT, and thus exocytosis of fibroblast-derived factors could not only fuel adjacent cancer cells but even comprise a feeding with resistance factors finally fostering PCa progression and therapy resistance. Thus, the loss of stromal CAV1 rather than the gain of epithelial CAV1 accounts more decisively for (radiation) therapy failure.

Differential CAV1 levels within PCa cells for example, were shown to impact on androgen receptor signaling. The androgen receptor-expressing LNCaP (and 22Rv1) PCa cells used in the present study, were known to have low endogenous CAV1 levels. Androgen receptor low or deficient cell lines (e.g., PC3 and DU145) in contrast expressed high CAV1 levels. Whereas downregulation of the androgen receptor did not impact on CAV1 expression levels, a further reduction of CAV1 in LNCaP cells inhibited androgen receptor expression and genomic activity, strongly suggesting that CAV1 could modulate rapid, non-genomic androgen receptor signaling at the plasma membrane (38). Here we speculate that RT-dependent increases in HSP27 phosphorylation levels could increase (androgen-independent) androgen receptor signaling by displacing other HSPs (e.g., HSP90) from the cytosolic complex with the androgen receptor subsequently squiring the receptor into the nucleus modulating target gene (especially EMT gene) expressions (**Figure 8**). CAV1 was already suggested to function as an androgen receptor co-regulator at advanced tumor stages (39, 40). Androgen stimulations usually can induce androgen receptor translocation to the cell membrane in a CAV1-dependent manner as reduced CAV1 levels were shown to decrease androgen receptor membrane localization (41). Mechanistically androgen receptor binding was achieved by CAV1 palmitoylation of the cysteine residues (41). Membrane-association in turn was shown to potentiate androgen receptor transcriptional activities by activating HSP27 further indicating that disruption of androgen receptor membrane translocation could represent a potential strategy for improving PCa therapy (42). Moreover, the metastatic spread as well as progression to castration-resistant PCa was already linked to increasing expression levels of HSP27 (43). Thus targeting CAV1-dependent HSP27 signaling potentially by SRC kinase inhibition represents a promising (androgen receptor disrupting) therapeutic strategy to limit PCa progression and moreover to sensitize PCa to RT. Accordingly, we showed here that a combined treatment with the SRC inhibitor dasatinib did not affect HSP27 phosphorylation but efficiently reduced total HSP27 levels either in LNCaP PCa cells with low endogenous CAV1 levels, effects that were accompanied by radiosensitization.

CAV1 as structural organizer generally regulates plasma membrane microdomain formations and thus, modulates signaling pathways to promote tumor and progression and potentially therapy resistance (44). In normal cells, CAV1 is predominately localized in the small microdomains called caveolae, specialized lipid rafts that are enriched in sphingolipids and cholesterol. Malignant prostate epithelial cells at advanced stages were shown to lack plasma membrane caveolae despite increasing CAV1 levels, based on the lack of cavin-1/polymerase I and transcript release factor (PTRF), a requisite component of caveolae. CAV1 is localized then in so called CAV1 scaffolds, non-caveolar homo-oligomerizations domains (36). PC3 PCa cells for example were shown to harbor high levels of non-caveolar CAV1 while lacking PTRF, and introducing PTRF in turn inhibited anchorage-independent growth, reduced *in vivo* tumor growth and metastasis (45). Likewise, fostered CAV1 but not PTRF expressions increased anchorage-independent growth of LNCaP and 22Rv1 cells (45). Conclusively, non-caveolar CAV1 promotes invasion and metastasis of PCa, while PTRF-induced caveolae formation reduced CAV1-dependent metastasis. Accordingly, we show here that even CAV1-overexpressing LNCaP cells lack PTRF and thus potentially caveolae-mediated signaling, corroborating the findings that the invasion capabilities of CAV1 WT LNCaP PCa cells were CAV1 scaffold-dependent.

An overexpression of CAV1 usually leads to a significant increase in overall levels of major lipid classes, particularly cholesterol (46). The increasing cholesterol content of cancer cells results in a more condensed, less fluid state with the increased potential of forming (larger) raft domains, even increasing the plasma membrane order (44). These processes are decisively regulated by CAV1. CAV1 binds cholesterol, thereby altering the solubility of membranes and regulating the formation of cholesterol-enriched micro-domains that even could cause depletion of cholesterol from other domains, and reduce the amount of accessible cholesterol (47). RT, and even increased cellular stress in general, affect the organization of these plasma membrane cholesterol-enriched micro-domains. By directly activating acid sphingomyelinase (ASMase), for example, and thus the increased generation of plasma membrane ceramide, RT induces the spatial reorganization of plasma membrane lipids, finally resulting in large ceramide-enriched domains (also termed large lipid rafts, LLP) as effective signalosomes (48, 49). Cholesterol is thereby displaced—at least partially—by ceramide and thus, the association of cholesterol with CAV1 is decreased. Lipid membranes were shown to generate two distinct types of condensed GM1 gangliosides (sialic acid-containing oligosaccharides covalently attached to ceramide lipids)-containing microdomains: rapidly formed but short-lived GM1 clusters that are formed in ceramide-rich domains and are predominantly involved in apoptosis, and long-lasting high-density GM1 condensations in the liquid-ordered phase, namely raft-like regions accounting for proliferation-promoting signaling (50, 51). As a more permanent plasma membrane modification, the latter one might account for signalosome-amplified signaling events upon CAV1 being present within the plasma membrane. Accordingly, CAV1 was previously shown to critically regulate this ASMase/ceramide-mediated response to RT, finally leading to increased RT resistance in CAV1-expressing PCa cells (25). However, no real shift to a more radio-resistant phenotype upon CAV1 expression was detectable here, which is most likely based on the fact that LNCaP cells already bear a quite resistant phenotype compared to other PCa cells (13, 16). Accordingly, only very low apoptosis and cell death levels (<5%) could be observed following irradiation. At all, the classically used two-dimensional cell cultures for *in vitro* analyses have many limitations, such as changes in cell morphology and polarity, which in turn impact on the cellular behavior (52, 53). As an approximations to the *in vivo* situations were additionally used an matrix-embedded 3D spheroid culture model to investigate the CAV1-dependent three-dimensional growth of LNCaP cells with differential CAV1 expression levels, and following the response to RT. It has to be noted that 48 h post RT and/or combined SRC inhibitor treatment, no real reduction in spheroids growth could been estimated for respective spheroids. We attributed that to the fact that the spheroid cultures investigated here were rather short as compared to previous investigations using PCa spheroids in combination with RT (53, 54). Respective red-fluorescent nuclear and chromosome counterstain using propidium iodide in turn clearly showed that CAV1 WT LNCaP spheroids contained less dead cells (48 h) following RT indicating a gain in RT resistance.

### CAV1-Dependent Regulations of the PCa Cell Metabolism Contribute to the RT Response

Most solid cancers were characterized by enhanced glucose uptake and associated increased glycolysis relative to oxidative phosphorylation yielding pyruvate and lactic acid even at aerobic conditions, a phenomenon known as the Warburg effect. However, PCa metabolism turned out to be unique as the metabolism here does not undergo the ‘Warburg glycolytic switch’ (55–57). While normal prostate epithelial cells mainly use glucose oxidation to finally synthesize and secrete citrate, malignant prostate epithelial do not show increased aerobic glycolysis (55). Instead, increased *de novo* lipid synthesis can be observed within the early phases of PCa with steadily increasing expression levels of lipogenic enzymes throughout PCa progression (56, 57). An increasing glucose metabolism to meet the requirements of increasing energy demands was then suggested for advanced, particularly castration-resistant PCa (58). Here we showed now that introducing either wildtype or mutant CAV1 had only minor effects on mitochondrial respiration rates in LNCaP cells, but increased respective glycolysis levels indicating higher energy demands. Particularly, the metabolic demands of CAV1 P132L LNCaP cells were found to be increased. The somatic mutation of CAV1 herein, a single point mutation within the transmembrane domain converting proline 132 to leucine (P132L), significantly reduced plasma membrane localization of CAV1 (59). Due to the fact that CAV1 P132L predominately can be found intracellularly, close to the perinuclear region and not at the plasma membrane, this mutant has become a valuable tool for studying miss-trafficked CAV1 (59, 60). CAV1 P132L LNCaP cells showed significantly less plasma membrane signaling platforms. At the same time, a decrease in cellular cholesterol was observed, which fits to the metabolic alterations, namely the increases in glycolysis to fulfill the respective energy demands, presumably caused by the reduced lipids and cholesterol utilization within CAV1 P132L LNCaP cells. The increasing energy demands for a fostered PCa cell growth further turned out to be based on a serine-threonine kinase AMP-activated protein kinase (AMPK)-mediated metabolic switch resulting in increased glycolysis rates together with an increase in glucose and fatty acid oxidation (61). Indeed, in patients, activation of AMPK correlated with PCa progression, further suggesting that AMPK might support PCa cells to survive under adverse nutritional conditions (62). Accordingly, the activity of AMPK was shown to be enriched in metastatic tumors compared to primary tumors (63). We detected increasing AMPK levels acting as metabolic sensor following increased CAV1 levels, effects that could efficiently be limited by SRC inhibitor treatment. Thus, targeting epithelial CAV1, particularly inhibiting the gain in advanced PCa, maybe the better therapeutic strategy for eliminating cancer cells because tumor starvation may additionally be induced.

### Targeting Stromal Fibroblasts for Improving the Response to RT

On the contrary, targeting fibroblastic CAV1 or more precisely stabilizing CAV1 levels in stromal fibroblasts could be a promising additional anti-cancer strategy, presumably concerning radiosensitization. Particularly SRC inhibition is suggested to enhance the efficacy of conventional therapies as the tumor stroma would even be beneficially targeted. The SRC inhibitor dasatinib was already shown to reverse the phenotype of CAFs to a phenotype comparable to that of normal fibroblasts, including reduction of proliferation rates (64). Even the metastasis-promoting actions of tumor fibroblasts were completely reversed by dasatinib treatment (65). However, in the complex *in vivo* situation, the effect of dasatinib seemed to be less efficient than suggested by corresponding *in vitro* studies using classically two-dimensional cultured PCa cells. Accordingly, no difference in overall survival and treatment failure of dasatinib was further estimated in patients with bone metastatic castration-resistant PCa (66). We even could not show any beneficial effects of SRC inhibition when LNCaP PCa cells were co-cultured with CAV1-expressing fibroblasts as 3D spheroids, a CAV1 stromal-epithelial state resembling more the early stages of PCa. Rather it seemed that co-applied RT itself could enhance the pathological drift from a tumor-suppressive to a tumor-promoting stroma. As a result, the efficiencies of applied treatments were limited by co-cultured fibroblasts in 3D spheroids. RT-induced changes within stromal fibroblasts generally come along with declining stromal CAV1 (**Figure 8**). The loss of stromal CAV1 in turn was accompanied with increased (stromal) TAGLN levels, an observation that was shown to account for a more activated and reactive nature of the respective tumor stroma (13, 16). Corroborating findings identified TAGLN as one (of two) proteins with the highest increase in expression in prostate CAFs (along with THY1/CD90 cell surface antigen) compared to non­malignant prostate fibroblasts (67). The loss of CAV1 within the tumor stroma was accompanied with a loss of stromal PTRF, effects that also correlated with higher Gleason scores, reduced relapse-free survivals, and thus poor outcomes (68, 69). Moreover, PTRF-deficient stromal cells increased the lipid contents of adjacent PCa cells, and promoted invasion and metastasis, strongly suggesting that stromal fibroblasts significantly foster an aggressive phenotype of PCa potentially by stromal feeding (69). Both, uptake of exogenous from the local microenvironment and also endogenous lipids, can be utilized through the fatty acid β-oxidation pathway, a pathway that is predominately used by non-glycolytic cancers, such as PCa (70, 71). The loss of stromal CAV1 upon PCa progression was further shown to be accompanied with a metabolic switch to aerobic glycolysis yielding lactate and pyruvate metabolites to fuel neighboring cancer cells and thus increasing their ATP production (36, 72). Rescued CAV1 expression levels within stromal fibroblasts in turn inhibited the cancer cells metabolism (73, 74). Thus, beside the intrinsic factors of cancer cells that determine the various metabolic pathways prevailing in respective cancer cells to fulfill the increasing energy demands, extrinsic factors derived from adjacent stromal fibroblasts were shown to alter cancer metabolism in a CAV1-depentend manner (75). SRC inhibition particularly by dasatinib could negatively impact on the situation as dasatinib was shown to increase the secretion of CAV1 in stromal cells (76), which potentially could foster the loss of stromal CAV1. Low CAV1 expression levels in stromal cells in turn caused alterations the respective lipid metabolism leading to an increased need for glucose that is fulfilled by enhancing aerobic glycolysis (77, 78). We corroborated here that irradiated fibroblasts exhibited an (CAV1-dependent) increased metabolic potential, an effect that was not affected by dasatinib treatment. Thus, activated fibroblasts/CAFs with low CAV1 levels are predestinated to fuel adjacent cancer cells. The CAV1-dependent metabolic coupling between stromal and cancer cells, finally achieving a stromal cell support of ‘energetic’ metabolites for the generation of further ATP in cancer cells is supposed to occur *via* the so-called exosomes (79). Especially the increased secretion patterns of lysosomal proteins such as ASMase, arylsulfatase A and more specifically of lysosomal-associated membrane proteins already indicated higher lysosomal exocytosis activities in more reactive (CAV1-deficient) fibroblasts (13, 16). The higher (stromal) secretion of ASMase in turn could foster the formation of ceramide-enriched membrane platforms on tumor cells, and thus allowing associated receptors to cluster here finally altering the cells signaling in terms of promoting resistance (80, 81). Finally, the CAV1-dependent stromal support of PCa cells was shown to comprise a feeding with resistance factors, e.g., apoptosis inhibiting proteins (13, 16).

In conclusion, the loss of stromal CAV1 rather than the gain of epithelial CAV1 accounts more decisively for RT therapy failure. Thus, targeting fibroblasts and/or limiting fibroblast activation, potentially by limiting the loss of stromal CAV1 seems to be the overarching strategy to inhibit the resistance-promoting signals of the tumor stroma.

## Data Availability Statement

The original contributions presented in the study are included in the article/**Supplementary Material**. Further inquiries can be directed to the corresponding author.

## Author Contributions

AW, JK, PM, LB, and DK performed experiments. AW and DK analyzed results and made the figures. CH and PM provided materials. VJ and DK designed the research. AW and DK wrote the paper. All authors contributed to the article and approved the submitted version.

## Funding

This work was supported by grants from the DFG (GRK1739/2), the BMBF (02NUK047D), and the Brigitte und Dr. Konstanze Wegener-Stiftung.

## Conflict of Interest

The authors declare that the research was conducted in the absence of any commercial or financial relationships that could be construed as a potential conflict of interest.

## Publisher’s Note

All claims expressed in this article are solely those of the authors and do not necessarily represent those of their affiliated organizations, or those of the publisher, the editors and the reviewers. Any product that may be evaluated in this article, or claim that may be made by its manufacturer, is not guaranteed or endorsed by the publisher.
