# Supplementary material for: Stromal Fibroblasts Counteract the Caveolin-1-Dependent Radiation Response of LNCaP Prostate Carcinoma Cells
Source: Front Oncol. 2022 Jan 26;12:802482. doi: 10.3389/fonc.2022.802482 (PMC8826751; doi:10.3389/fonc.2022.802482)
Supplement: Supplementary file 1 [file Presentation_1.pdf]

Supplementary Material

Supplementary Figures

Supplementary Figure S1

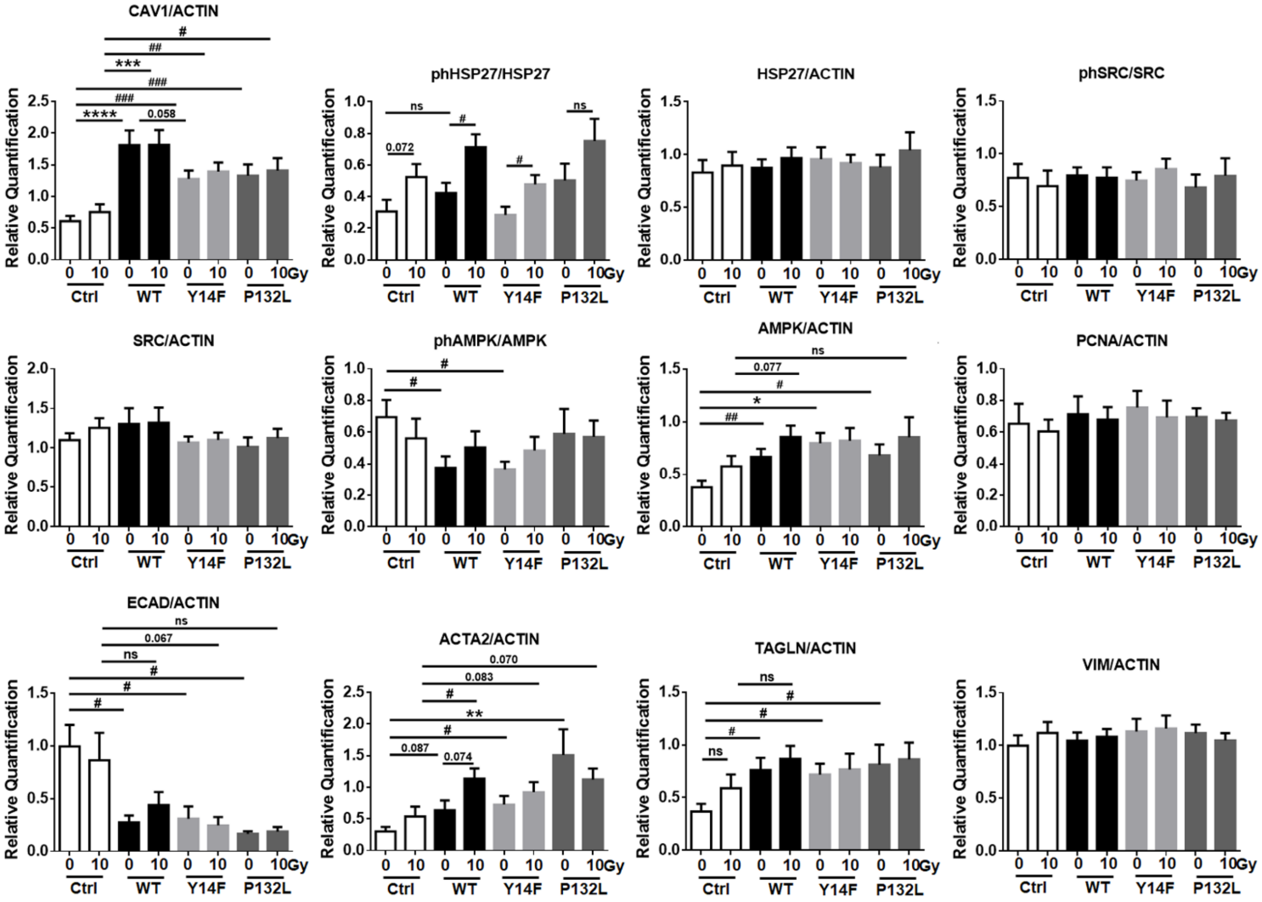

Densitometric quantifications of the Western blot signals presented in Figure 1A. Expression levels of the indicated proteins were analyzed in whole protein lysates of cultured LNCaP PCa cells stably overexpressing the introduced CAV1 variants (wildtype CAV1, WT; phosphorylation-deficient CAV1, Y14F; proline-132-to-leucine substituted CAV1, P132L) with or without radiation treatment (48 hours after RT with 10Gy) using Western blot analysis. Empty vector transduced LNCaP cells served as controls [Ctrl (CAV-)]. Respective blots are shown in Figure 1A. For quantification blots were analyzed by densitometry and respective signals were related to beta-actin (at least n=4 for each group). When the phosphorylation status was determined the obtained phospho-specific signals were related to the signals of the total protein. P-values indicate: *$p \leq 0.05$, **$p \leq 0.01$, ***$p \leq 0.005$, ****$p \leq 0.001$ by one-way ANOVA followed by post-hoc Tukey's comparison test and additionally by unpaired (two-tailed) t tests depicted as # $p \leq 0.05$, ## $p \leq 0.01$, ### $p \leq 0.005$.

Supplementary Figure S2

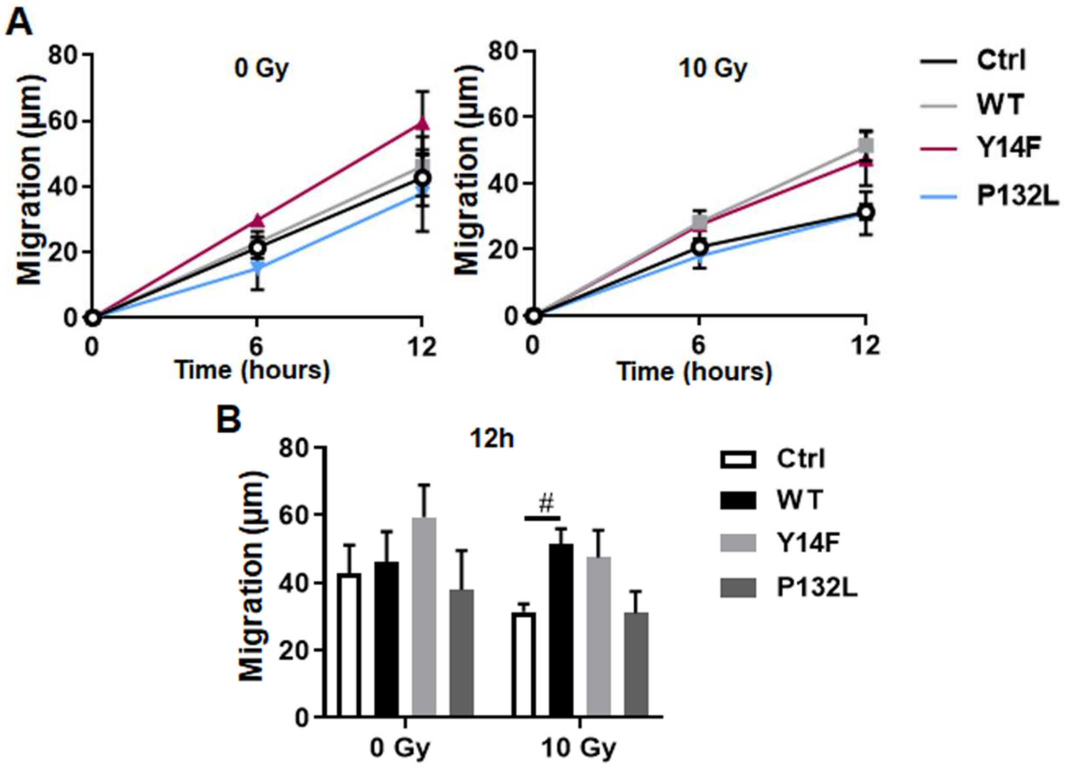

Ectopic expression of CAV1 does not affect migrations of LNCaP cells under non-irradiated conditions while the migration of CAV1 WT LNCaP PCa cells was enhanced following RT. LNCaP PCa cell migrations were investigated 48 hours after irradiation with 10Gy following introduction of a thin wound in confluent monolayers by scratching with a pipette tip. Wound closure was determined for the different CAV1 variants (wildtype CAV1, WT; phosphorylation-deficient CAV1, Y14F; proline-132-to-leucine substituted CAV1, P132L) as well as of control (Ctrl) LNCaP cells by measuring the migration distance (wound closure) after 6 and 12 hours. Wound closures were related to the distance of the introduced wound and the migrated distance was calculated. (**A**) The migration capabilities of indicated LNCaP cultures following 6 and 12 hours (0Gy and 10 Gy) were summarized. (**B**) The wound closure after 12 hours following (0 and 10Gy) were additionally summarized in a bar diagram. Data are shown as means ± SEM of 3-5 independent experiments. P-value indicates # $p \leq 0.05$ by unpaired (two-tailed) t-test (compared to Ctrl).

Supplementary Figure S3

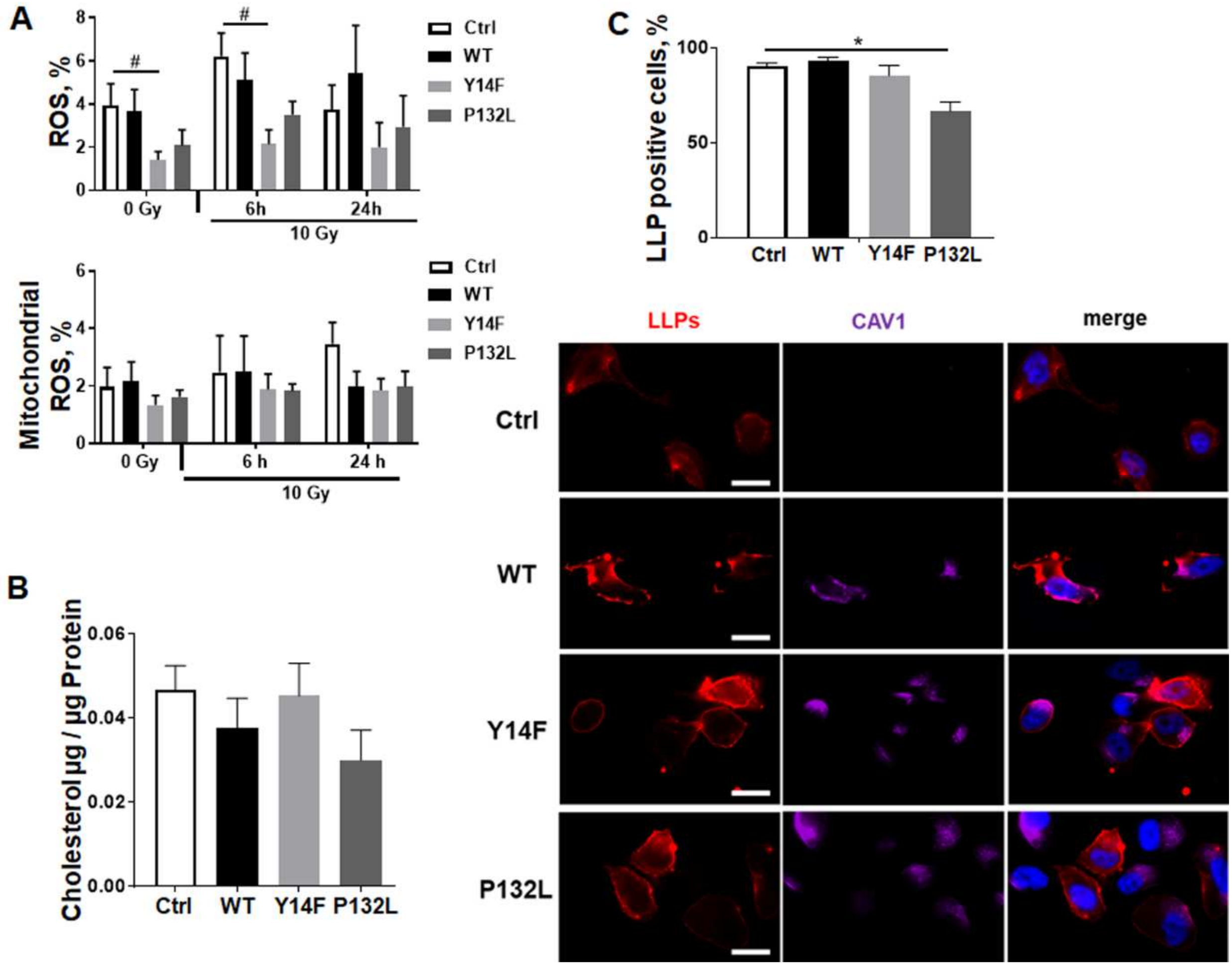

Reduced cholesterol levels are accompanied by reduced signaling platforms at the plasma membrane in CAV1 P132L LNCaP cells. (**A**) RT-induced cellular and mitochondrial stress was analyzed in CAV1 WT, CAV1 Y14F, and CAV1 P132L LNCaP PCa cells as well as in control (Ctrl) cells by measuring ROS production at 6 and 24 hours of post RT using dihydroethidium staining, and using a red mitochondrial superoxide indicator in combination with flow cytometry analyses. Data are shown as mean values ±SEM of 3-6 independent experiments. P-values indicate # $p \leq 0.05$ by unpaired (two-tailed) t-test. (**B**) Total cholesterol concentrations were determined and obtained cholesterol levels were related to respective protein concentrations (µg cholesterol per µg protein). Data are shown as means ±SEM of 3-5 biological replicates. (**C**) The steady state distributions of large lipid platforms (LLP) at the plasma membrane in CAV1 WT, CAV1 Y14F, CAV1 P132L as well as of control (Ctrl) LNCaP PCa cells were visualized using Cholera toxin subunit B (red), which binds ganglioside GM1. CAV1 staining are depicted in purple and nuclei were stained with Hoechst 33342 (blue). Representative photographs are shown. Quantification of LLP formation was done by counting GM1 positive cells. (n=3-5 with 50 cells per condition and per independent experiment, mean +/-SEM). Statistical analysis was performed with one-way ANOVA followed by post-hoc Tukey's comparison test (* $p \leq 0.05$). Scale bar: 20 µm.

Supplementary Figure S4

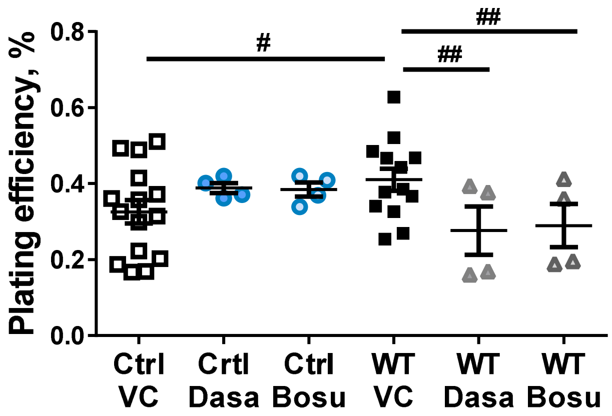

Treatments with the SRC inhibitors dasatinib and bosutinib reduce the increased plating efficiencies of CAV1-expressing LNCaP PCa cells. LNCaP PCa cells stably overexpressing the introduced WT CAV1 as well as control (Ctrl) LNCaP cells with low endogenous CAV1 levels were cultured in normal growth media supplemented with the SRC inhibitors dasatinib, bosutinib or vehicle control (VC) 2 h prior radiation treatment with 0 Gy or 10 Gy. Plating efficiencies were determined after 10 days following plating of low cell numbers (500 cells per 35 mm dish, plated in triplicates). Symbols depict individual values from different independent experiments. P-values indicate # $p \leq 0.05$, ## $p \leq 0.01$ by unpaired (two-tailed) t-tests (compared to VC).

Supplementary Figure S5

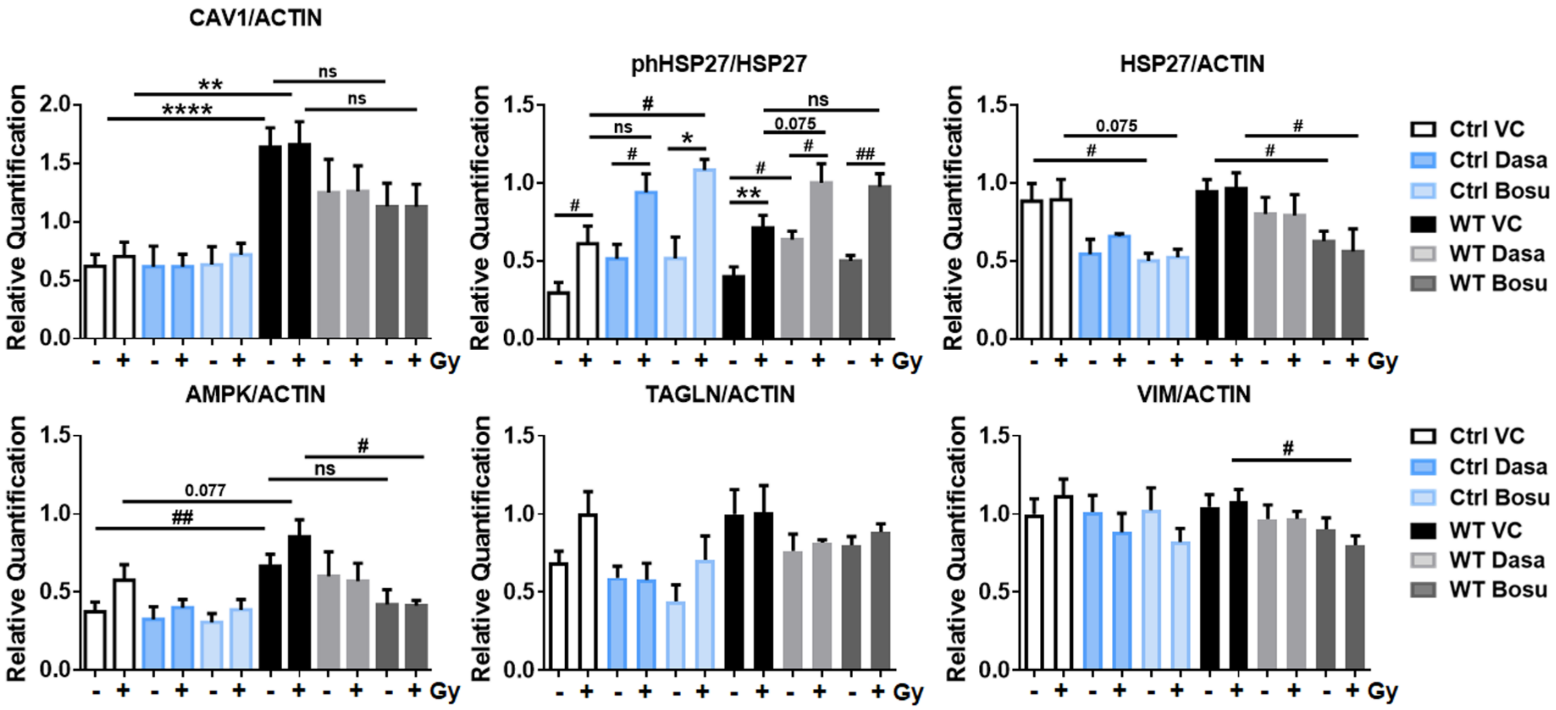

Densitometric quantifications of the Western blot signals presented in Figure 4D. Expression levels of the indicated proteins were analyzed in whole protein lysates of cultured LNCaP PCa cells stably overexpressing the introduced CAV1 variants (wildtype CAV1, WT; phosphorylation-deficient CAV1, Y14F; the transmembrane affecting proline-132-to-leucine substituted CAV1, P132L) with or without radiation treatment (48 hours after RT with 10Gy) using Western blot analysis. Empty vector transduced LNCaP cells with low endogenous CAV1 levels served as controls [Ctrl (CAV-)]. Respective blots were shown in Figure 4D. For quantification blots were analyzed by densitometry and respective signals were related to beta-actin (at least n=4 for each group). When the HSP27 phosphorylation status was determined the obtained phospho-specific signal was related to the signal of total HSP27 protein. Phosphorylated AMPK levels could not be quantified as respective signals following SRC treatment were too low. P-values indicate: *$p \leq 0.05$, **$p \leq 0.01$, ****$p \leq 0.001$ by one-way ANOVA followed by post-hoc Tukey test and additionally by unpaired (two-tailed) t tests depicted as # $p \leq 0.05$, ## $p \leq 0.01$.

Supplementary Figure S6

Supplementary Figure S6

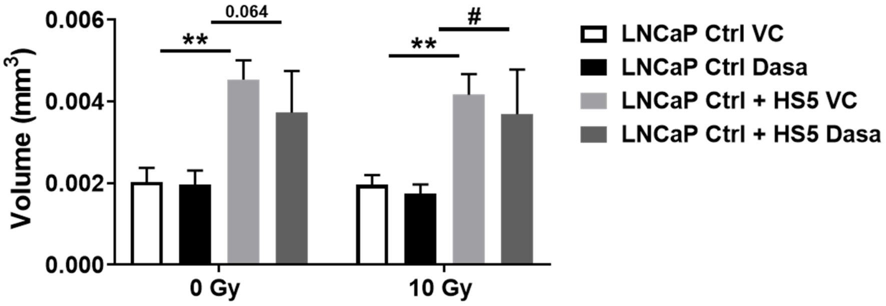

Stromal fibroblasts increase the growth of LNCaP spheroids upon co-culture. LNCaP control (Ctrl) PCa cells expressing low endogenous CAV1 levels were cultured either alone or together with CAV1-expressing stromal fibroblasts (HS5) in hanging drops for 24 h. After formation of spheroids, cells were plated in growth factor-reduced Matrigel mixed with normal growth medium (1:2, v/v) supplemented with dasatinib or vehicle control and irradiated at 0 or 10 Gy. Spheroid growth was measured and the respective volumes were calculated 48 h post treatment. Graphs depict the mean from 3-8 independent experiments where at least 10 spheroids per condition each were measured. P-values indicate: ** $p \leq 0.01$ by one-way ANOVA with Tukey's multiple comparison post-test and additionally by unpaired (two-tailed) t test depicted as # $p \leq 0.05$.

Supplementary Figure S7

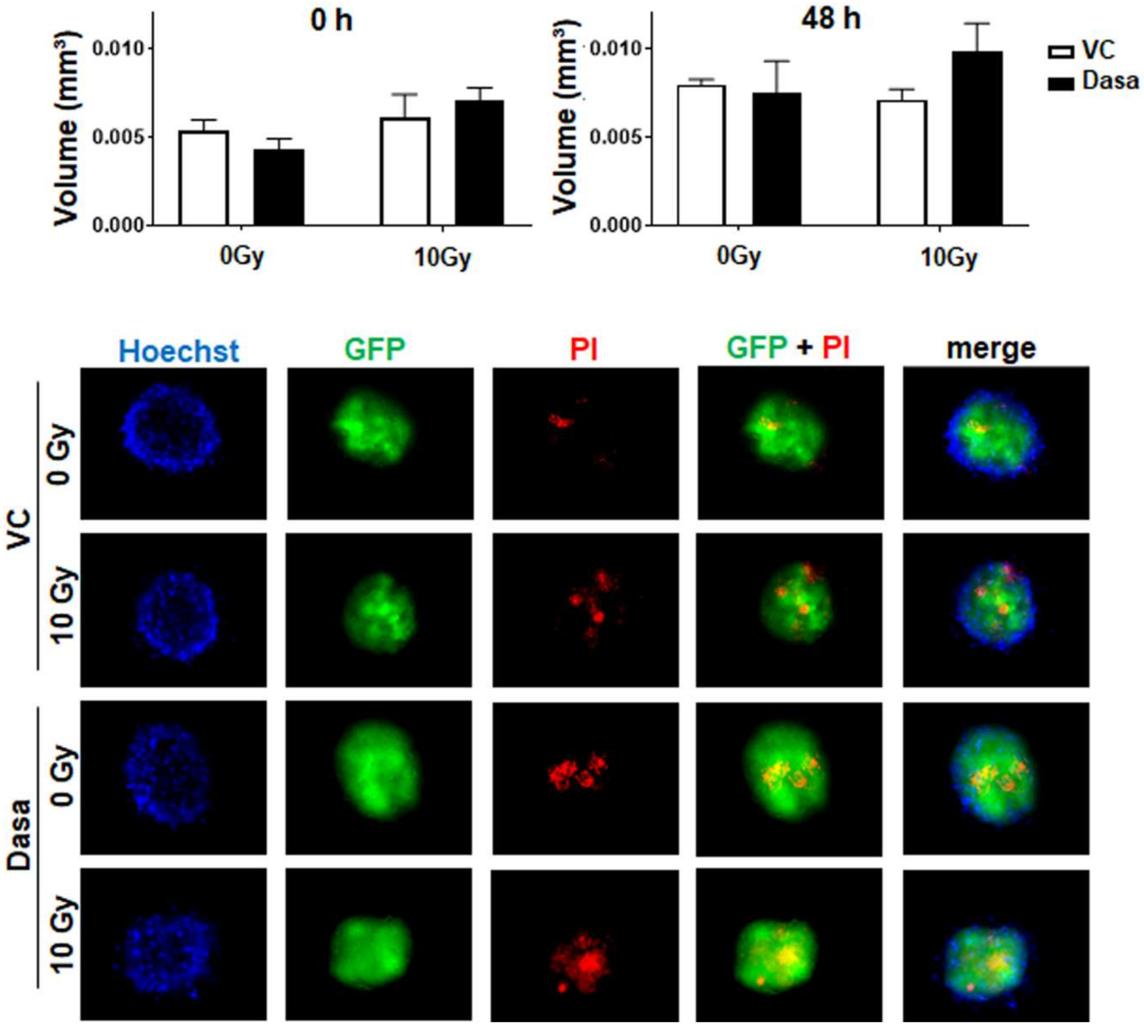

Spheroid co-cultures of (androgen responsive) 22Rv1 PCa cells and CAV1-expressing fibroblasts lack a response to RT either following combined dasatinib treatment. CAV1 expressing stromal fibroblasts (HS5) were co-cultured with 22Rv1 PCa cells (expressing low endogenous CAV1 levels) as spheroids in normal growth medium supplemented with dasatinib or vehicle control and irradiated at 0 or 10 Gy. Spheroid growth was measured and the respective volumes were calculated for 0 h and 48 h after irradiation. Data were summarized as mean +/- SEM as estimated in 3-5 individual experiments where at least 10 spheroids (per condition and per experiment) were measured. Cell death was analyzed afterwards by fluorescence microscopy using propidium iodide. Hoechst 33342 was used for nuclei staining. Representative fluorescent images from the individual experiments are shown (48 h time point).

Supplementary Figure S8

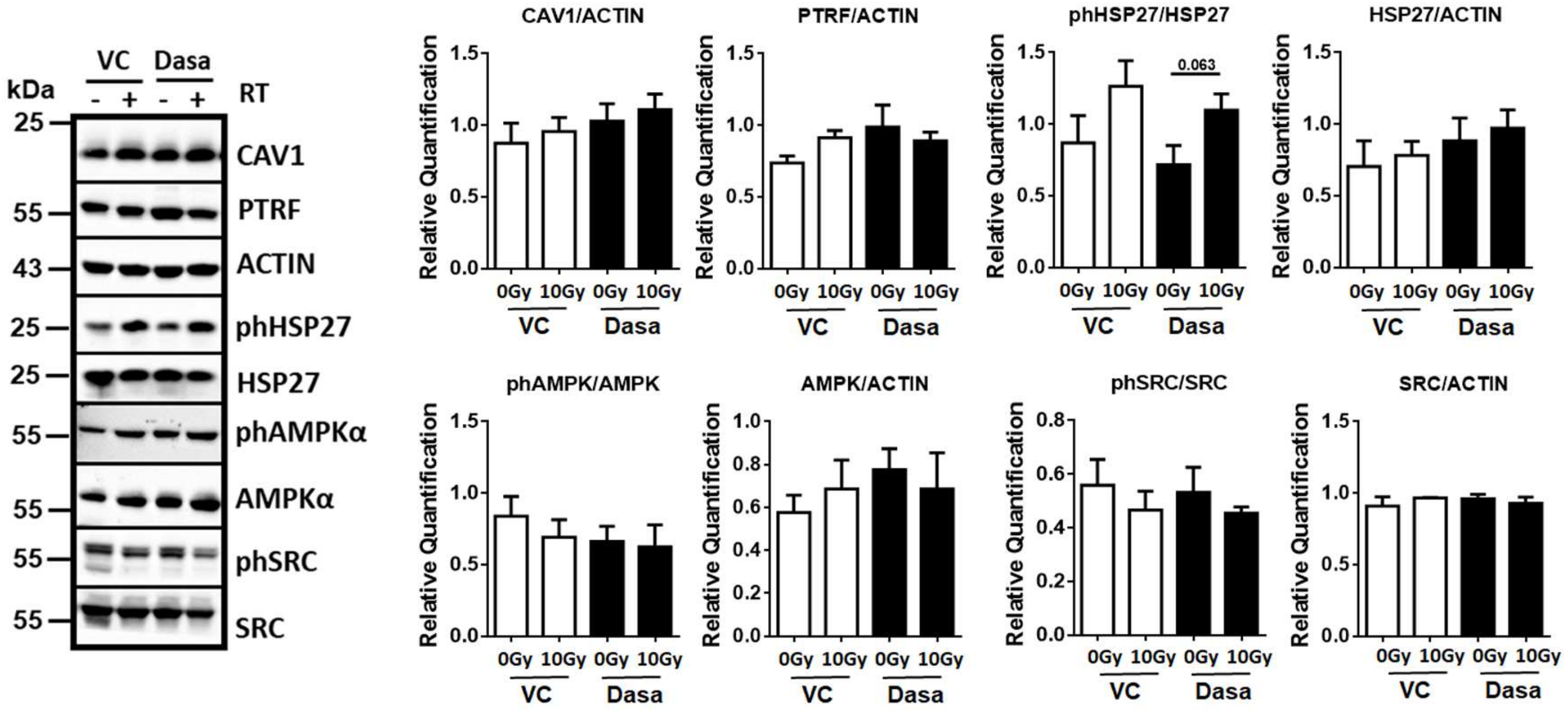

RT does not severely impact on SRC or AMPK signaling in stromal fibroblasts, nor in combination with dasatinib treatment, although HSP27 signaling trends to be increased. CAV1 expressing stromal fibroblasts (HS5) were cultured as monolayers with or without radiation treatment (0Gy and 10Gy) in the presence of dasatinib or vehicle control and irradiated at 0 or 10 Gy. Expression levels of the indicated proteins were analyzed in whole protein lysates of respective cultures 96 hours after RT with 10Gy using Western blot analysis. Representative blots are shown. For quantification blots were analyzed by densitometry and the respective signal was related to beta-actin (n=3-4 for each group). When the phosphorylation status was determined the obtained phospho-specific signal was related to the signal of the total protein.

Supplementary Figure S9

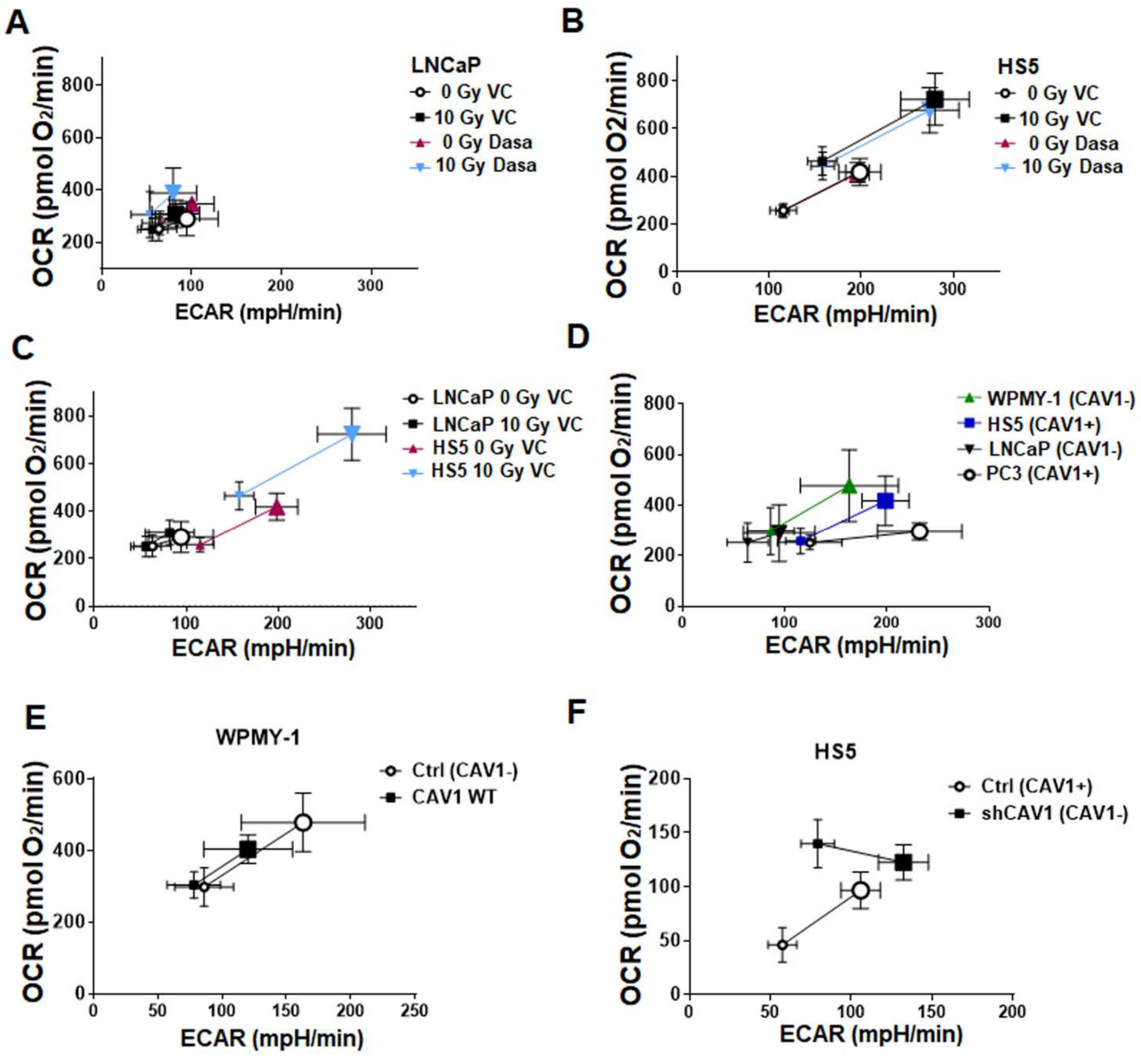

RT differentially impacts on cellular stress in stromal fibroblasts and malignant prostate epithelial cells as determined by cell metabolism alterations. The relative utilization of the two energy pathways mitochondrial respiration and glycolysis, as estimated by oxygen consumption rates (OCR) and extracellular acidification rates (ECAR) over time, in LNCaP PCa cells with low endogenous CAV1 levels (**A**) and stromal fibroblasts (**B**) were summarized upon both baseline and stressed conditions following RT treatment (0Gy and 10Gy) in the presence of dasanitib or vehicle control (VC) 24 hours post treatments. Basal levels (small symbols) of untreated cells and stressed levels (enlarged symbols) after the addition of oligomycin, FCCP, rotenone and antimycin A are shown. Whereas in LNCaP cells mitochondrial oxidative phosphorylation and glycolysis levels were

not affected by RT and/or dasatinib treatment, RT caused increasing energy demands of fibroblasts as revealed by increased mitochondrial respiration and glycolysis levels. (**C**) The response to induced energy demands, known as the cell’s metabolic potential, is higher in stromal fibroblasts than in LNCaP cells, effects that were associated with an activated, more reactive fibroblast phenotype that was not affected upon SRC inhibition. (**D**) The metabolic potential of CAV1-expressing fibroblasts (HS5), stromal prostate fibroblasts with low endogenous CAV1 expression levels (WPMY-1), CAV1-expressing PCa cells (PC3), and LNCaP PCa cells with low endogenous CAV1 expression levels are shown. Stromal fibroblasts showed an increased metabolic potential compared to PCa cells, with additionally increased levels in fibroblasts expressing low endogenous CAV1 levels (WPMY-1). CAV1-expressing PCa cells even showed an increased metabolic potential. Thus, in an advanced PCa situation characterized by low CAV1 levels within the stromal compartment and high CAV1 levels within the malignant epithelial cells, targeting of stromal fibroblasts could be the ultimate goal for CAV1-modulating therapeutic strategies. Particularly stabilizing fibroblastic CAV1 would limit the increased metabolic potential of prostate (WPMY-1) fibroblasts with low endogenous CAV1 levels, as suggested by a decreased metabolic potential of upon CAV1 overexpression (**E**). Similarly, a decreased metabolic potential finally limiting the support of adjacent PCa cells was estimated in CAV1-expressing (HS5) fibroblasts as compared to respective cells following CAV1-silencing (shCAV1) (**F**).

Supplementary Figure S10

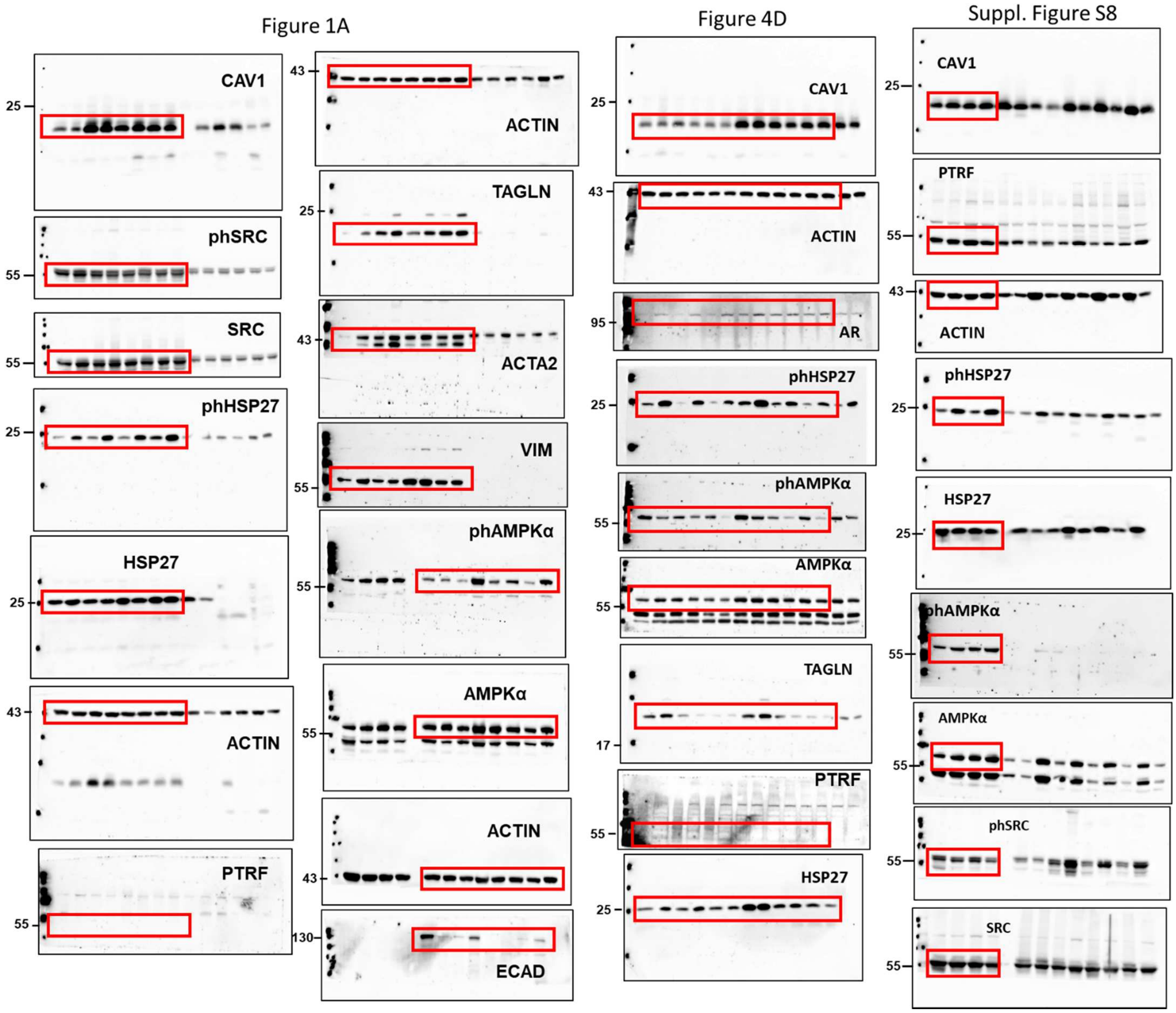

Full gels of cropped gels (emphasized by a red rectangle) as shown in Figure 1A, 4D, and Supplementary Figure S8. Equal protein amounts were loaded. Beta-actin was included as a loading control.
